# Decomposition-Induced Change in Stranded Megafauna: Decoupling Postmortem Impacts from the Isotopic Niche

**DOI:** 10.1021/acs.est.6c03636

**Published:** 2026-07-10

**Authors:** Philip M. Riekenberg, Lonneke L. IJsseldijk, Mardik Leopold, Andrea Gröne, Marcel T.J. van der Meer

**Affiliations:** † Department of Marine Microbiology & Biogeochemistry, 10209 NIOZ Royal Netherlands Institute for Sea Research, University of Georgia Center for Applied Isotope Studies, PO Box 59, Den Hoorn 1790AB, The Netherlands; ‡ Division of Pathology, Department of Biomolecular Health Sciences, Faculty of Veterinary Medicine, Utrecht University, Yalelaan 1, Utrecht 3854 CL, The Netherlands; § Wageningen Marine Research, Wageningen University and Research, Ankerpark 27, Den Helder 1781 AG, The Netherlands

**Keywords:** decomposition, stable isotope, amino acid, nitrogen, carbon

## Abstract

Diet throughout an animal’s lifetime can be reconstructed using stable isotope analyses of tissues with different turnover times, yet postmortem changes affecting these measurements remain understudied. Here, we investigate bulk δ^13^C, δ^15^N, and δ^34^S, and amino acid δ^13^C and δ^15^N values from multiple tissue types sampled from stranded harbor porpoises across a range of initial decomposition states. Decomposition caused a consistent decline in δ^13^C values across tissues and amino acid classifications, indicating anaerobic microbial fermentation as a dominant metabolism driving changes in early decomposition. δ^15^N and δ^34^S values were largely stable, suggesting limited microbial transformation of nitrogen and sulfur pools prior to more advanced decomposition. Tissue-specific isotopic differences were larger for both bulk and amino acids values, likely driven by metabolic differences between tissue types as well as differences in elemental turnover from diet. Our findings highlight predictable isotopic carbon shifts during early decomposition and call for caution when interpreting stable isotope data from potentially degraded samples. Moderately decomposed marine mammal tissues reflect diet if tissue-specific discrimination is applied, and amino acid selection impacts both nitrogen and carbon interpretations due to varying differences between metabolic classifications.

## Graphical abstract

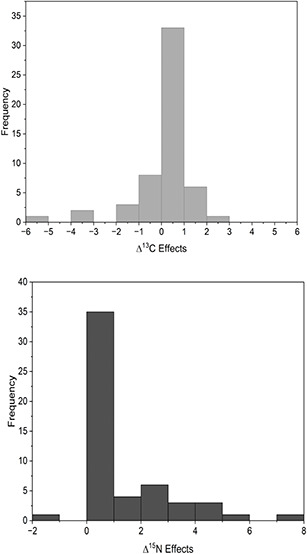

## Introduction

Decomposition due to microbial degradation is a universal process that affects all tissues after death and has held scientific interest for centuries.^1–3^ Putrefaction alters the chemical makeup of tissues as it develops, fundamentally changing the underlying molecular and elemental composition that ecologists and forensic anthropologists rely upon to reconstruct dietary composition, habitat use, origin, or physiological state for individuals prior to death.^4^ Cell autolysis causes tissue degradation due to the release of hydrolytic enzymes as cellular membranes lose their integrity, and putrefaction develops as anaerobic bacteria proliferate in the gastrointestinal tract, causing bloating and then liquefaction.^5,6^ Cellular hydrolysis results in the export of materials (e.g., gases or liquids) that are expected to undergo kinetic fractionation,^7^ leaving the tissue they originated from with a higher stable isotope value after exporting a product that has a lower stable isotope value than the original substrate.^4^ However, postmortem impacts from decomposition on isotopic values vary widely between elements (e.g., carbon, nitrogen, and sulfur) and molecules (e.g., amino acids), indicating that other drivers of isotopic change are being observed as decomposition develops (Table 1).

**Table 1:** Review of Existing Studies Using Stable Isotope Measurements to Examine Microbial Decomposition Effects on Tissue^a^

Paper #	system targeted	animal	length of time	δ^13^C Effect	δ^15^N Effect	δ^34^S Effect	C/N
1	Mammalian	Beaver	300 days	0‰	2–5‰ increase, more in active tissue		increased for gut, lungs, and muscle
2	Mammalian	Ringed seal	0 to 256 days	0.3‰ ↑	0.7‰ ↑		no change
2	Fish	Trout	0 to 256 days	0.3‰ ↓	0.3‰ ↑		no change
2	Shark	Greenland shark	0 to 256 days	1.1‰ ↓	0.4‰↑		0.2↑
3	Mammalian	Human	20 days	0‰ for skin, 1‰ ↑ for muscle	0.8‰ ↑ for skin, 2.5‰ ↑ for muscle		
3	Mammalian	Pig	12 days	0‰ for skin and muscle	0‰ for skin, 0.5‰ ↑ for muscle		
4	Mammalian	Finless porpoise	stranded vs bycaught	0.4‰ ↓	1.6‰ ↑		0.2 to 0.4↑
5	Reptile	loggerhead hatchlings	∼3 days dead in nest	0.8‰ ↓	1‰ ↓		
6	Insect	Drosophila	10 days	0.8‰ ↓	0.4‰ ↑		
7	Mammalian	Killer whale	14 days	1‰ ↑ skin, 0.4↓ blubber	7‰↑ skin, 1‰ ↑ blubber		
8	Mammalian	Gray whale	stranded vs subsistence	0.7‰ ↑ muscle, 1.2‰ ↑ skin non-IE	0.9‰ muscle↑, 0‰ skin		
9	Mammalian	Striped dolphin	62 days	0‰ skin, 0‰ muscle	0‰ skin, 0‰ muscle		no change
9	Reptile	loggerhead	62 days	0‰ skin, 0‰ muscle	0‰ skin, 0‰ muscle		no change
10	Fish	Grouper, Rabbitfish	5 days	0.2 to 0.4‰ ↑	0.6‰ ↑		
10	Crustacea	Mantis shrimp, crab	5 days	0.2 to 0.4‰ ↑	0.5‰ ↑		
10	Mollusc	Gastropod, Bivalve	5 days	0.3‰ ↓	no effect		
11	Mammalian	Bottlenose dolphins	11 days	0.5‰ ↓ liver, no effect	0.5‰ ↑ liver, no effect		Increased for liver, no effect otherwise
11	Mammalian	Bottlenose dolphins	fresh vs decomp state	3–4‰ ↓ liver and skin, no effect muscle	no effect		
11	Mammalian	Manatees	11 days	0.5‰ ↓ liver, no effect	0.5‰ ↑ liver, no effect		Increased for liver, no effect otherwise
11	Mammalian	Manatees	fresh vs decomp state	5–7‰ ↑ liver,	no effect		
12	Fish	Pink Salmon	4 days water 8 in air	1‰ ↑ skin, fin, muscle, no effect for scale	no effect		
13	Insect	Chironomid	8 days	0.3‰ ↑ food items	5‰ ↑ microbial enrichment to food items		
14	Mammalian	Pine Marten	8 to 18 months	↓0.2–1.4‰ bulk, –9.4–2‰ amino acids	4‰ ↑, –2.6–5.4‰ amino acids		
14	Mammalian	Pine Marten	8 to 18 months	1–2‰ ↓, –9.4–2‰ amino acids	3–5‰ ↑, –2.6–5.4‰ amino acids		mixed between inoculations
15	Fish	Salmon	225 days	up to 6‰ ↓, lipid release into fluids	4‰ ↑, in fluids released		
16	Mammalian	Human	32 days	no effect	no effect		
17	Mammalian	Human	22 to 1140 days	no effect	0–1.1‰ ↑		
18	Mammalian	Harbor Porpoise	unknown	0.5‰↓ Bulk, 0.7‰↓ amino acid	no effect	no effect	

	tissue type															
Paper #	M	Li	Bl	C	B	Lu	Ha	He	Fat	GT	S	W	F	Sc	Reference	Muscle
1	X	X	X		X	X	X	X	X	X					Keenan and DeBruyn 2019	Liver
2	X														Yurkowski et al. 2017 open vial	Blood
2	X														Yurkowski et al. 2017 open vial	Collagen
2	X														Yurkowski et al. 2017 open vial	Bone
3	X										X				Miles 2024	Lung
3	X										X				Miles 2024	Hair
4	X														Furuyama et al. 2020	Heart
5											X				Frank et al. 2012	Fat
6												X			Ponsard and Amou	Gut tissue
7									X		X				Burrows et al. 2014	Skin
8	X										X				Horstmann-Dehn et al. 2011	Whole
9	X										X				Payo–Payo et al. 2013	Fin
9	X										X				Payo–Payo et al. 2013	Scale
10	X														Perkins et al. 2018	
10	X														Perkins et al. 2018	
10	X														Perkins et al. 2018	
11	X	X									X				Cloyed et al. 2023	
11	X	X									X				Cloyed et al. 2023	
11	X	X									X				Cloyed et al. 2023	
11	X	X									X				Cloyed et al. 2023	
12	X										X		X	X	Peiman et al. 2021	
13															Goedkoop et al. 2006	
14				X											Turban-Just and Schramm 1998	
14				X											Balzer et al. 1997	
15												X			Wheeler et al. 2014	
16				X											Hoke et al. 2019	
17							X								Saul et al. 2021	
18	X	X	X	X											This study	

a Tissue types reported are Muscle (M), Liver (Li), Blood (Bl), Collagen (C), Bone (B), Lung (Lu), Hair (Ha), Heart (He), Fat, Gut Tissue (GT), Skin (S), Whole Organism (W), Fin (F), and Scale (Sc).

Stable isotope values from consumer tissues are used to identify resource and habitat use,^8,9^ spatial placement during movements across wide-ranging ecosystems,^10,11^ origin,^12,13^ and to reconstruct physiological condition across an animal’s lifetime.^14^ Consistent differences between stable isotope values for dietary resources in an ecosystem allow for tracing dietary use for consumers (e.g., bulk or amino acid δ^13^C values^15–18^ ) and consistent increases in diet-consumer relationships (trophic discriminations) give rise to the ability to track ecosystem trophic structure (bulk and amino acid δ^15^N^19,20^ ). These techniques rely on the consistent temporal integration of material from the consumer’s diet^21,22^ and accounting for potential alterations of incorporated values (e.g., taphonomic or preservation impacts^23,24^ ). Removal or addition of elemental materials during preservation^25–27^ or alterations to elemental compositions during postmortem decomposition^4^ can bias stable isotope reconstructions of diet or physiology, leading to increased uncertainty in modeling efforts to assign dietary reliance or spatial occupancy. Previous work has found that alterations from decomposition range from −6‰ to +7‰, with δ^13^C frequency grouping around no effect and δ^15^N values generally increasing if effects were observed (Figure 1; *n* = 54 from 17 studies;^4,7,28–43^ Table 1), with studies having two approaches to examining decomposition: 1) taking tissue from individuals and aging it in appropriate *ex situ* settings, or 2) examining a range of individuals decomposing *in situ* to identify how decomposition arises. However, these previous efforts have not included δ^34^S values and present only limited decomposition impacts on amino acid δ^13^C and δ^15^N values (e.g., only available from Turban-Just and Schramm,^40^ Balzer et al.^41^), where soil bacteria shifted bone collagen amino acid values by −2.9‰ and +5.8‰ for δ^13^C and δ^15^N, respectively, across 8 to 18 months of *ex situ* incubation

**Figure 1:**
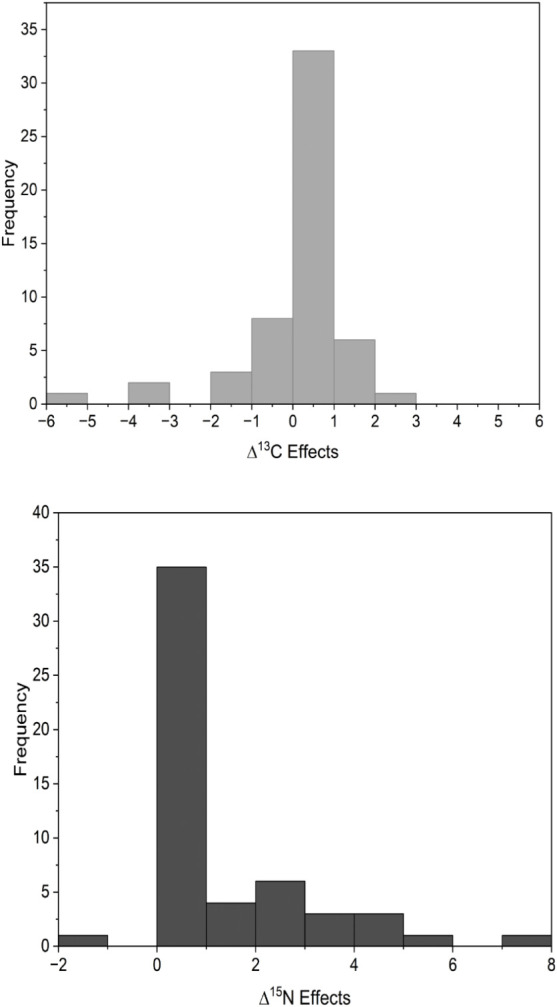
Frequency distribution of Δ^13^C and Δ^15^N values for decomposition effects (‰) found in existing studies. n = 54 from the 17 studies listed in Table 1.

Research on the feeding ecology of fully aquatic marine mammals is challenging, as financial, logistical, and ethical constraints limit the extent to which free-living individuals can be observed, sampled, and resampled.^44,45^ These limitations highlight the importance of making use of opportunistic samplings from marine mammal strandings from organized long-term stranding national observation networks to further inform conservation and management practices. The application of stable isotope analyses to tissues obtained from tissue archives of stranded marine mammals provides a valuable opportunity for investigating long-term dietary patterns in a statistically robust manner.^46^ Marine mammals are usually mid- to apex predators and may govern the structure of the ecosystem that they reside in (e.g., top-down pressure^47^). To use tissues from stranded animals, analyses must account for turnover times—the time necessary for the complete replacement of the elemental composition of tissue (δ^13^C, δ^15^N, or δ^34^S) from their diet—to identify changes in resource use across an animal’s lifetime.^48^ By using multiple tissue types, it becomes possible to reconstruct dietary resource use at multiple time points prior to the death of the animal to capture periods of time where dietary stress or starvation may become apparent.

In stranded marine mammals, decomposition has often already progressed to some degree by the time the carcass is recovered, and samples are stored for further analysis. Following the best practices guidelines put forward by the European Union cetacean management bodies, ASCOBANS (Agreement on the Conservation of Small Cetaceans of the Baltic, North East Atlantic, Irish and North Seas) and ACCOBANS (Agreement on the Conservation of Cetaceans of the Black Sea, Mediterranean Sea, and the Contiguous Atlantic Area), stranded animals are graded across a range of decomposition condition codes (DCC) from extremely fresh (DCC1) to mummified or solely skeletal remains (DCC5).^49,50^ Under moderate to more advanced states of decomposition, tissues such as liver and muscle remain relatively preserved, and analyses can inform upon lifestyle prior to death, but other samples, such as blood, become impossible to reliably retrieve. Bone collagen is comparatively well-preserved through initial decomposition^51^ but reflects a mix of material incorporated 1–2 years prior to death due to lower rates of turnover for skeletal collagen.^52,53^ Targeting a tissue with a shorter turnover time (e.g., blood or fluid from the liver) that is reliably recovered from initial to moderate decomposition conditions will help to reconstruct feeding and foraging grounds leading up to the last weeks prior to death. This additional snapshot closer to the time of death may provide vital metabolic insight into whether the animal experienced longer-term food scarcity or a shorter, more intense period of starvation that was not recorded in tissues with longer turnover times (e.g., muscle, collagen, skin).

Here, we compare the isotopic composition of multiple tissue types (blood, liver, muscle, and collagen) from harbor porpoises (*Phocoena phocoena* *)* to identify differences in bulk carbon, nitrogen, and sulfur isotopic values, as well as amino acid carbon and nitrogen stable isotopic values. Harbor porpoises are the most abundant marine mammals in the North Sea and strand regularly,^54^ making their tissues readily available for archiving and long-term storage as part of a dedicated postmortem sampling program. We identify differences in isotopic values across multiple tissue types and decomposition states and examine whether blood-like fluid taken from the liver can be used as a useful postmortem proxy for isotopic values from blood in harbor porpoises.

## Methods

Since 2008, harbor porpoises have been necropsied as a routine part of the monitoring effort at the Faculty of Veterinary Medicine, Utrecht University, The Netherlands.^55^ Individuals in this study were limited to decomposition conditions 1 to 3, indicating very limited to moderate decomposition, and at least prior to the liquefaction of the softest tissues, which makes recovery of the liver difficult due to putrefaction. Dorsal muscle (*Longissimus dorsi*), whole blood, liver, and rib (5th rib on the left side) were selected from 86 porpoises necropsied between 2009 and 2021, with samples stored frozen (−20 °C) at Utrecht University prior to transport and analyses at the NIOZ Royal Netherlands Institute for Sea Research. Here, samples were partially thawed, residual remnant tissue removed (ribs), and a subsample was collected (0.5 mL of venous blood by glass pipet, 1–5 g of muscle and liver, ∼1 g of liver fluid, 50–100 g of bone), then freeze-dried prior to grinding. Liver fluid was isolated while liver samples were still frozen, as the liquid was increasingly difficult to select without the inclusion of liver tissue as samples thawed. Collagen was extracted from 1 cm blocks cut ∼5 cm inward on the rib from the attachment point to the sternum after undergoing surface removal using a rotary tool with a sanding wheel. 300 mg of prepared bone was demineralized in 2 N HCl for 24 to 36 h at 5 °C, with the acid replaced once after ∼8 h. The remaining tissue was then rinsed with bidistilled water 5 times to neutrality, lipid extracted using a 3/2 v/v mixture of hexane/dichloromethane 3×, and dried under a stream of N_2_ at 40 °C. The demineralized remnant was then washed 3× with bidistilled water prior to adding 10 mL of bidistilled water and acidifying with 50 μL of 0.1 M HCl and heating for 12–16 h at 70 °C. After cooling, the extract was filtered through a tightly packed, solvent-extracted cotton plug, frozen, and lyophilized for 24–36 h.

### Stable Isotope Analysis

For the analysis of bulk δ^13^C, δ^15^N, and δ^34^S, 2–3 mg of freeze-dried, homogenized, and nonlipid extracted material was loaded into tin capsules. Samples were analyzed with an Elementar Vario Isotope Cube elemental analyzer connected to an Isoprime VisION isotope ratio mass spectrometer at NIOZ (Texel). Analytical precision for CNS analysis is ±0.1‰ for δ^13^C, ±0.2‰ for δ^15^N, and ±1.2‰ for δ^34^S using internal reference standards across a 3-point scale for each isotopic range, which are scaled against the standards NBS-22, IAEA-N1, IAEA-S2, and S3, which are reported against Vienna Pee Dee Belemnite, atmospheric N_2_, and Canyon Diablo Troilite. Stable isotope ratios are expressed in the δ notation as units per mil (‰), calculated as

$$\delta_{C,\;N,\;\mathrm{or}\;S}=\left(R_{\mathrm{sample}}-R_{\mathrm{standard}}\right)/R_{\mathrm{standard}})-1$$

where

R = ^13^C/^12^C, ^15^N/^14^N, or ^34^S/^32^S.

Samples were rerun after lipid extraction if the C/N ratio was higher than 4.5, where ∼2 mL of a 3/2 dichloromethane/hexane (V/V) was added in a glass vial to 2–3 mg of sample and immediately vortexed for 30 s. The solvent was then removed via a glass pipet and new solvent was added and vortexed (2×) or until the removed solvent was clear. The sample material was then dried at 40 °C under a stream of N_2_ until completely dry and then packed into tin capsules for analysis.

Preparation of the derivatives for the analysis of δ^13^C and δ^15^N values for individual amino acids used 1.5–3 mg of freeze-dried and homogenized tissue that was hydrolyzed overnight (12–18 h) in 200 μL of 6 N HCl at 110 °C. The hydrolysates were cooled, lipid-extracted, filtered through a 0.45 μm PTFE centrifuge filter, and stored for up to 3 months at −20 °C prior to further processing. Two different derivatization pathways were used to prepare amino acids for analysis via gas chromatography-combustion isotope ratio mass spectrometry: *n*-acetyl isopropyl esters (NAIP) for δ^13^C analysis^56^ and n-pivaloyl/isopropyl esters (NPiP) for δ^15^N analysis.^57–59^ For the NAIP esters, 10 μL of the hydrolyzate was spiked with norleucine and dried under N_2_ at 40 °C to dryness, then isopropylated using a mixture of isopropanol and acetyl chloride (3/2, v/v) and heated to 110 °C for 2 h. The product was then cooled, evaporated to dryness (N_2_, 40 °C), and DCM (300 μL) was added and evaporated to dryness (2×). The AA i-propyl esters were then acylated using a mixture of acetyl anhydride, triethylamine, and acetone (1/2/5, v/v/v) and heated at 60 °C for 15 min. The derivative was then cooled, evaporated to dryness, and DCM (300 μL) was added and evaporated to dryness (2×; N_2_, 40 °C). Preparation of NPiP esters is described in detail in^59^ and used 190 μL of hydrolyzate after subsampling for amino acid δ^13^C values. After preparation, the dried derivatives were resuspended in DCM (NAIP) or ethyl acetate (NPiP) and analyzed via gas chromatography flame ionization detector to quantify concentration to target for analysis via gas chromatography-combustion-isotope ratio mass spectrometry (GC-C-IRMS). Derivatized samples were analyzed for δ^13^C and δ^15^N for individual amino acids separately, in duplicate, using a Trace 1310 gas chromatograph coupled to a Delta V Advantage isotope ratio mass spectrometer through an IsoLink II interface. Samples were standardized against the internal reference standard spike of norleucine and two standard mixes: one with 15 amino acids to account for combustion and derivatization effects and one with 7 amino acids provided by Arndt Schimmelmann to account for day-to-day machine variation in measurement across the corresponding ranges on per mil scales using the correction workflow presented in Yarnes and Herszage^60^ and described in greater detail in Riekenberg, van der Meer, and Schouten.^59^ Details about the temperature ramps, flow rates, and column materials used are provided in Supplemental 1.

For δ^13^C values for amino acids, we grouped them into essential (Leu, Phe), nonessential (Ala, Asp), and conditionally essential (Glu, Pro), and for δ^15^N values for amino acids, we grouped them into trophic (Ala, Glu), source (Phe), and metabolic (Gly, Thr). These groupings reflect the relative preservation of isotopic values through metabolism, with relatively less fractionation occurring in essential and source groupings and larger fractionation associated with nonessential and trophic groupings. Conditionally essential and metabolic groupings have fractionations that are variable, given the physiological condition affecting the metabolism of the individual. All amino acid δ^15^N values are normalized against lysine δ^15^N values to account for baseline differences in N supporting the ecosystem during the times when dietary materials were incorporated into each tissue type.

Ethical review and approval were not required for the animal study because the animals described in this study were free-living harbor porpoises that died of natural causes or were euthanized on welfare grounds, and not for the purpose of this or other studies. No consent from an animal use committee was required, as the animals described in this study were not used for scientific or commercial testing. Consequently, animal ethics committee approval was not applicable to this work.

### Data Analysis

Bulk δ^13^C, δ^15^N, δ^34^S, amino acid δ^13^C and δ^15^N values were examined using Levene’s homogeneity of variance test prior to analysis using 2-way ANOVA with the variables tissue type (blood, liver, muscle, collagen, liver fluid) and decomposition condition (freshly dead, DCC1-DCC2; moderate decomposition, DCC3), with Tukey’s honest significant difference test run post hoc. Bulk Δ^13^C, Δ^15^N, Δ^34^S values, and amino acid Δ^13^C and Δ^15^N values between tissue types, were examined using Levene’s homogeneity of variance test prior to analysis using 2-way ANOVA. We applied a comparison against blood based on the expectation that amino acids derived from the diet enter the body through the intestine into the hepatic portal and are then further metabolized via different metabolic pathways dependent on tissue type. Comparisons between tissue types (liver-blood, muscle-blood, collagen-blood, and liver fluid-blood) and decomposition condition codes were included as design variables for the 2-way ANOVAs with Tukey’s honest significant difference test run post hoc with α = 0.05. Levene’s homogeneity of variance tests and 2-way ANOVAs were performed using OriginPro 2024 (10.1.5.132). To further explore differences between design variables (decomposition state, tissue type) among groupings of amino acids, we applied analysis of variance simultaneous component analysis (ASCA) to both normalized amino acid δ^15^N and δ^13^C data sets. ASCA is a multivariate ANOVA-style analysis designed to examine multivariate responses against one or more design variables. The ASCA in the HDANOVA package^61,62^ in R Studio (2023.09.1b494) and R (R Core Team 2024) does this by applying linear mixed models to provide two-dimensional relationships between predetermined experimental design variables and then applying principal component analysis to the least squared estimates to explore differences between design variables, with provided confidence ellipsoids. All tables supporting the statistical analyses are provided in Supplemental 2.

## Results

### Bulk Analyses

Bulk δ^13^C (‰) values are 0.5‰ lower for moderately decomposed samples (Figure 2A; F_1,114_ = 9.6, *p* = 0.003), with a higher value difference found for collagen than for liver, with blood, muscle, and liver fluid falling between those two values with overlapping distributions (Figure 3A; F_4,114_ = 4.7, *p* = 0.002), with all tissue types within ∼1‰ for median values. Δ^13^C values between tissue types are positive for collagen-blood (0.5‰) with the other tissue types having a negative difference (Figure 3B; F_3,91_ = 5.8, *p* = 0.001), with values of −0.3 to −0.4‰ that are comparable between decomposition states for tissue types. Bulk δ^15^N values are lower for muscle tissue than for all other tissue types (0.4–0.6‰; Figure 3A; F_4,114_ = 8.2, *p* < 0.001), and δ^15^N values are similar for decomposition conditions among tissues. Δ^15^N values are lower for muscle-blood (−0.9‰) than for any of the other tissue differences examined (0.1–0.2‰; Figure 3B; F_3,91_ = 9.9, *p* < 0.001), with similar values for decomposition conditions. Bulk δ^34^S values are lower for collagen than for all other tissue types (Figure 3A; F_4,114_ = 7.4, *p* < 0.001), and δ^34^S values are similar between decomposition states. Δ^34^S values are only different for collagen-blood (−2.8‰), with similar values for all other tissue comparisons (0.3–0.9‰; Figure 3B; F_3,91_ = 6.7, *p* < 0.001) and with similar values for decomposition states.

**Figure 2:**
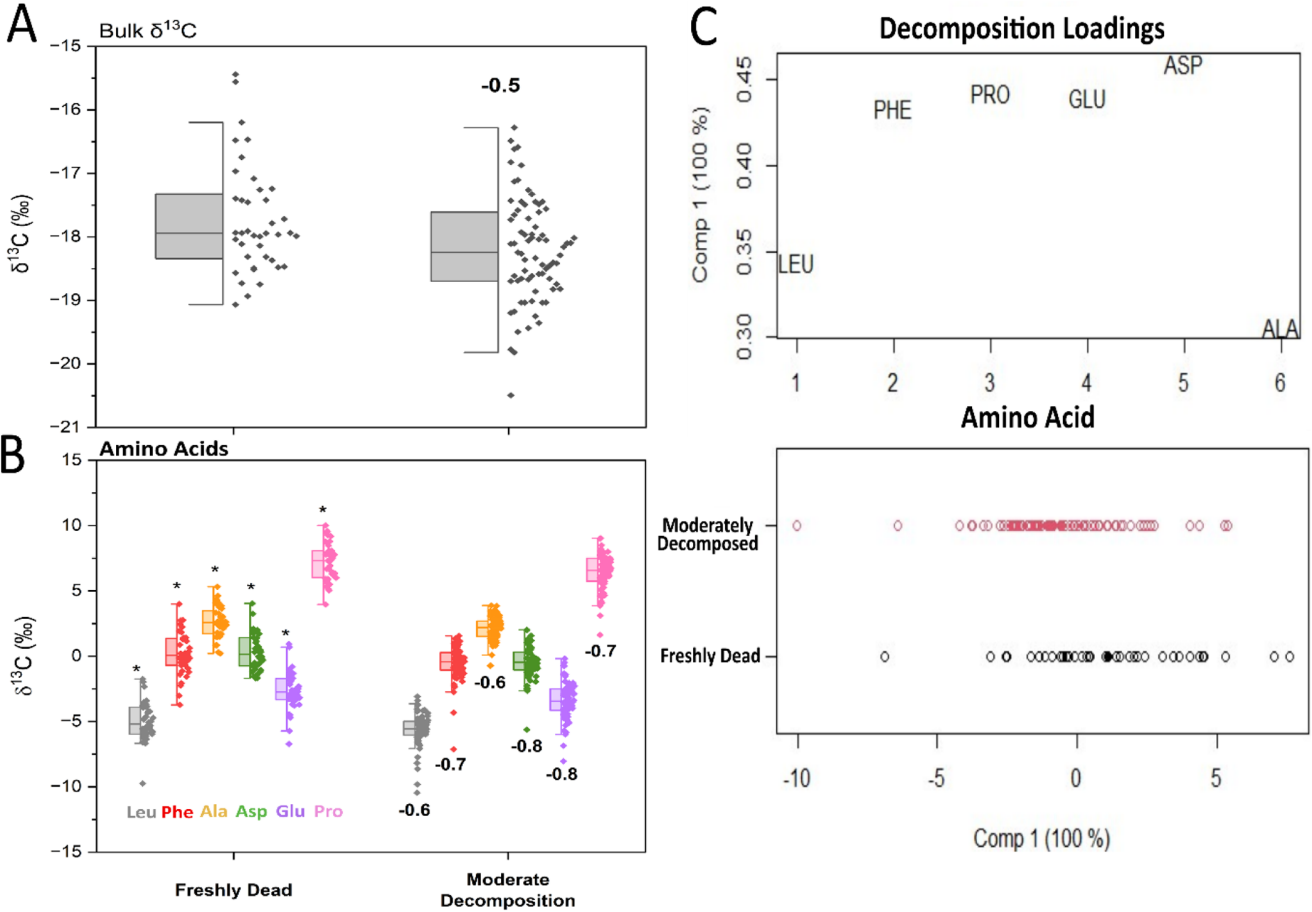
A) Bulk δ^13^C values, B) amino acid δ^13^C values, and C) ASCA loadings and differences between decomposition states for amino acid δ^13^C values from harbor porpoise tissues. Bold numbers are the values for the median ‰ differences between decomposition states. Boxes represent the 25 to 75% quartiles, whiskers are the 1.5 interquartile range, and the line indicates the mean value for each grouping. Asterisks indicate differences between decomposition states from post hoc Tukey’s honest significant difference at α = 0.05.

**Figure 3:**
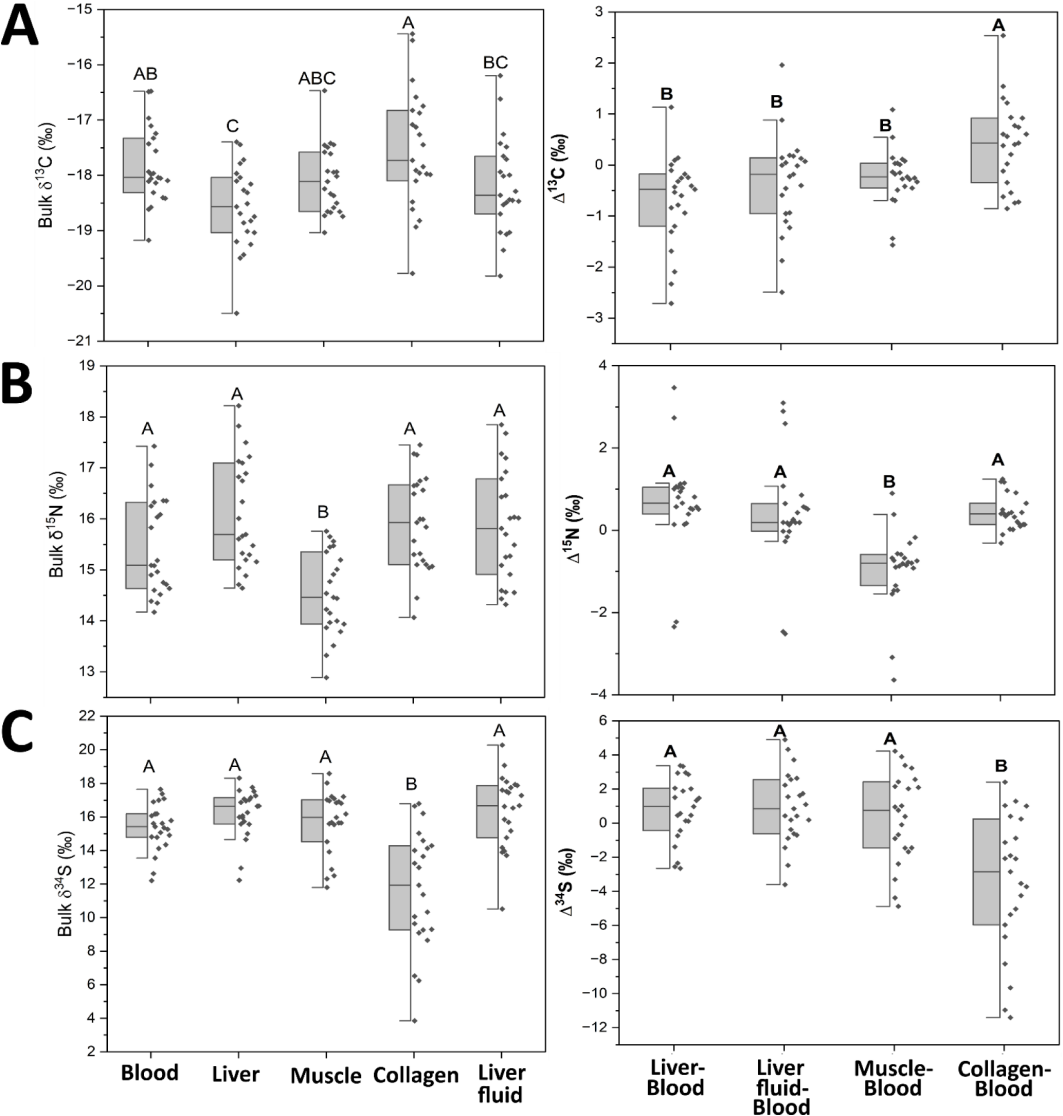
A) Bulk δ^13^C, B) δ^15^N, and C) δ^34^S values for the 5 different harbor porpoise tissue types and A) Δ^13^C, B) Δ^15^N and C) Δ^34^S values for liver, liver fluid, muscle, and collagen measured against whole blood. Boxes represent the 25 to 75% quartiles, whiskers are the 1.5 interquartile range and the line indicates the median value for each grouping. Letters indicate likewise groupings from post hoc Tukey’s honest significant difference at α = 0.05.

### Amino Acid Analyses

Amino acid δ^13^C values were normalized against the mean following best practices presented in Larsen, Taylor, Leigh, and O’Brien^16^ and Vane et al.,^63^ to compare the accumulated differences in biosynthetic pathways between tissue types and during decomposition. All amino acid δ^13^C values had lower values for moderate decomposition versus freshly dead, of ∼0.7‰ (Figure 2B; Supporting Information 2). We present amino acids grouped into essential, nonessential, and conditionally essential groups, dependent on the relative preservation of the amino acid during metabolism. For the essential amino acid leucine, there are no differences between tissue types for Tukey’s HOD, but the 2-way ANOVA indicates higher values for muscle and collagen versus liver fluid and blood, which are close to statistical significance (Figure 4A; F_4,114_ = 2.4, *p* = 0.051). For phenylalanine, blood δ^13^C values are higher than those for liver fluid, with liver, muscle, and collagen falling intermediate between (F_4,114_ = 4, *p* = 0.005). For the nonessential amino acid alanine, muscle and collagen δ^13^C values are higher than those for liver fluid, with blood and liver being intermediate between (Figure 4B; F_4,114_ = 5.4, *p* < 0.001). Aspartic acid δ^13^C values are higher for muscle versus blood and liver fluid, with collagen and liver being intermediate between (F_4,114_ = 5.1, *p* < 0.001). For the conditionally essential amino acid glutamic acid, δ^13^C values for muscle are higher than blood with liver, liver fluid, and collagen being intermediate between (Figure 4C; F_4,114_ = 3, *p* = 0.02). For proline δ^13^C values, blood and muscle are higher than liver and liver fluid, with collagen being intermediate between (F4,114 = 5.5, *p* < 0.001).

**Figure 4:**
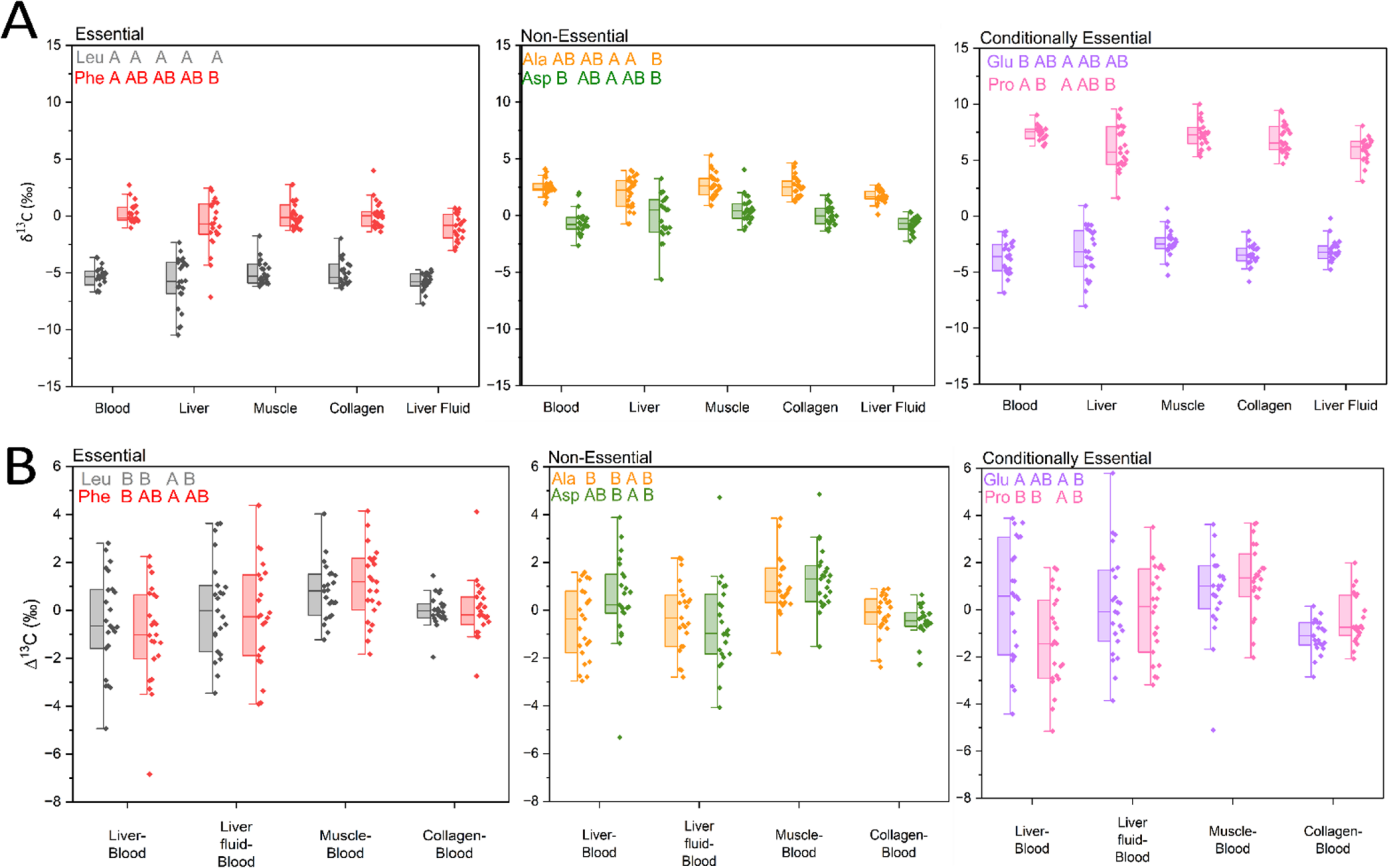
A) δ^13^C and B) Δ^13^C values for mean normalized essential (leucine, phenylalanine), nonessential (alanine, aspartic acid), and conditionally essential (glutamic acid, proline) amino acids for the tissue types and tissue comparisons in this study. Boxes represent the 25 to 75% quartiles, whiskers are the 1.5 interquartile range and the line indicates the median value for each grouping. Letters or asterisks indicate groupings from post hoc Tukey’s honest significant difference at α = 0.05 for comparison across tissue types and decomposition states for each normalized amino acid and tissue comparison.

Δ^13^C value differences between tissue types for the essential amino acid leucine are higher for muscle than for all other tissue types (Figure 4B; F_3,91_ = 7.6, *p* < 0.001). Phenylalanine Δ^13^C value is higher for muscle than for liver, with liver fluid and collagen intermediate between (F_3,91_ = 5.2, *p* = 0.002). For the nonessential amino acid alanine, the Δ^13^C value for muscle is higher than for all other tissue types (F_3,91_ = 7.6, *p* < 0.001). The aspartic acid Δ^13^C value is higher for muscle than for liver fluid and collagen, with liver being intermediate between (F_3,91_ = 10.4, *p* < 0.001). The conditionally essential amino acid glutamic acid has higher Δ^13^C values for liver and muscle than for collagen, with liver fluid intermediate between (F_3,91_ = 8.3, *p* = 0.005). Proline Δ^13^C values are higher for muscle than for all other tissue types (F_3,91_ = 8.4, *p* < 0.001). There are no differences in Δ^13^C values for the decomposition state across any of the amino acid tissue pairings.

Lysine δ^15^N values are higher for liver than for collagen, which are the highest and lowest tissue type values, with liver fluid, muscle, and blood being intermediate between the two values (supplemental Figure 5; F_4,114_ = 12.3, *p* < 0.001), and with no difference observed for decomposition state. δ^15^N values for the trophic amino acid glutamic acid are highest for collagen and lowest for liver and liver fluid, with muscle and blood at intermediate values between (Figure 5A; F_4, 113_ = 16.4, *p* < 0.001). Proline has a lower δ^15^N value for blood but equivalent values across all other tissue types (F_4, 113_ = 10.1, *p* < 0.001). Both the source amino acid phenylalanine and the metabolic amino acid glycine have higher δ^15^N values for collagen but equivalent values across all other tissue types (Figure 5A; Phe F_4,114_ = 27.7, *p* < 0.001; Gly F_4,114_ = 14.8, *p* < 0.001). For threonine, liver and muscle have lower δ^15^N values than blood and collagen, with liver fluid being intermediate between (Figure 5A; F_4,114_ = 9.4, *p* < 0.001). Glutamic acid is the only amino acid with higher δ^15^N values for moderately decomposed tissues (F_1,113_ = 7.7, *p* = 0.007).

**Figure 5:**
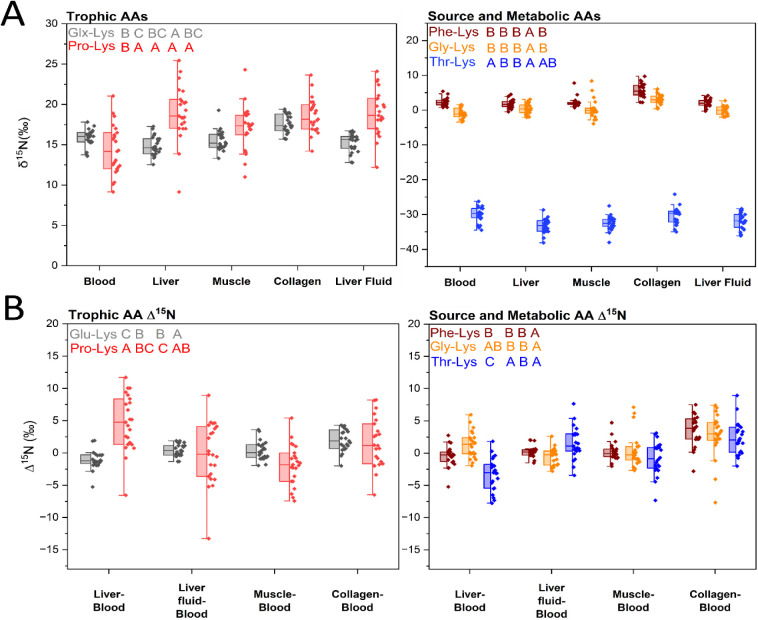
A) δ^15^N values and B) Δ^15^N values for lysine-normalized trophic (glutamic acid, proline), source (phenylalanine), and metabolic (glycine, threonine) amino acids for the tissue types and for the tissue types versus blood examined in this study. Boxes represent the 25 to 75% quartiles, whiskers are the 1.5 interquartile range, and the line indicates the median value for each grouping. Letters indicate likewise groupings from post hoc Tukey’s honest significant difference at α = 0.05 for comparison across and between tissue types for each normalized amino acid.

Δ^15^N values for the trophic amino acid glutamic acid between tissue types are higher for collagen than for liver (Figure 5A; F_3,91_ = 14.1, *p* < 0.001), with liver fluid and muscle falling intermediate between. For the trophic amino acid proline, liver Δ^15^N values are higher than for muscle (F3,91 = 10.3, *p* < 0.001), with collagen and liver fluid intermediate between. Δ^15^N values for the source amino acid phenylalanine are higher for collagen than for all other tissues, which had equivalent values (Figure 5B; F_3,91_ = 20.1, *p* < 0.001). Δ^15^N values for the metabolic amino acid glycine are higher for collagen than for either liver fluid or muscle, and liver is intermediate between (F_3,91_ = 5.4, *p* = 0.002). Δ^15^N values for the metabolic amino acid threonine are highest for collagen and liver fluid and lowest for liver, with muscle intermediate between (F_3,91_ = 21.1, *p* < 0.001). There are no differences in Δ^15^N values for the decomposition state for any of the amino acid tissue pairings.

For amino acid δ^13^C values normalized against their mean, both factors, tissue type (Figure 6B; SS = 152, VAR = 12%, *p* < 0.001) and decomposition status (Figure 3C; SS = 75, VAR= 6%, *p* < 0.001), explain significant differences between design variables. Exploring the loadings driving the observed differences, proline (0.53), phenylalanine (0.50), and alanine (0.43) are the main contributors to component 1 and glutamic acid (0.72) and aspartic acid (0.55) are the main contributors to component 2. For lysine-normalized amino acid δ^15^N values, only the factor tissue type explained significant variance (Figure 6A; SS = 1094, VAR = 34%, *p* < 0.001), with phenylalanine (−0.60) and glycine (−0.58) being the main contributors to component 1, and proline (0.78) and threonine (−0.55) driving contributions to component 2.

**Figure 6:**
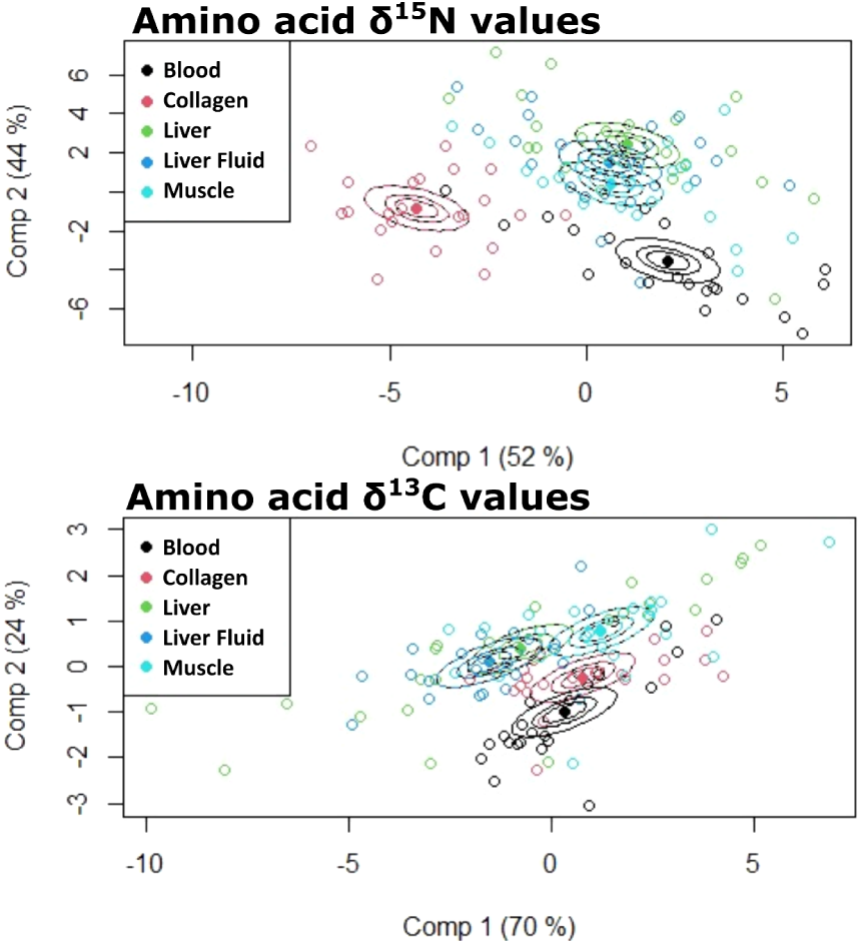
Analysis of variance simultaneous component analysis (ASCA) for A) lysine-normalized amino acid δ^15^N values and B) mean-centered amino acid δ^13^C values using tissue type and decomposition condition as grouping factors. Confidence ellipsoids represent 40%, 68%, and 95% uncertainty intervals scaled on data dispersion and sample size.

## Discussion

### Decomposition Effects on Carbon

Our analysis examining decomposition from a well-replicated range of stranded porpoises across a range of minimal to moderate decomposition states found differences in bulk (Figure 2A; −0.5‰) and individual amino acid δ^13^C values (Figure 2B; −0.7‰) as well as a consistent impact across a limited selection of amino acid types across multiple tissue types (Figure 1C; essential, nonessential, and conditional). Moderate decomposition appears to drive lower values for not only the bulk carbon pool but also for amino acid δ^13^C values from proteins across all tissue types. The uniformity of this process likely indicates a common impact during the development of putrefaction, potentially due to: 1) cellular hydrolysis, 2) consistent microbial fractionation among tissues, or 3) the use of lipids or loss of sugars to fuel anaerobic microbial processing, leaving microbially produced de novo amino acids in decomposing tissues with lower δ^13^C values than in materials from freshly dead animals. If the driving factor were associated with hydrolysis or solely decarboxylation^64^ during the breakdown of cellular materials, we would expect kinetic fractionation to dominate,^65^ where products have lower isotopic values and their substrate pool is left with a higher value. Instead, we observe decreased δ^13^C values for bulk and amino acids from partially decomposed tissues (microbes + exudates + tissue; DCC3; Figure 2) that represent the affected substrate pool. To account for these decreased values, we started to look for potential pools with: 1) lower δ^13^C values that are ubiquitous among tissue types, such as lipids (e.g., cell wall or storage), or 2) substrates with higher δ^13^C values that are rapidly removed and leave the substrate pool lower in δ^13^C value, which may be contributing to supporting predominantly anaerobic microbial metabolism as cells are no longer provided with oxygen from active respiration. As cell hydrolysis occurs during developing putrefaction, anaerobic bacteria that are typically part of the gut microbiome (i.e., *Clostridium* spp.) proliferate across tissue types as host immune systems cease to keep bacteria in check.^66,67^ During the development of moderate decomposition (DCC3), microbes initially proliferate in a relatively closed system that quickly becomes anaerobic within tissues and then the body cavity due to the cessation of circulatory respiration. *Clostridium* spp. typically metabolize hexose sugars into short-chain acids and subsequently solvents (e.g., butanol, acetone, isopropanol) via fermentation in the intestines (Guo et al., 2020).^200^ Microbial fermentations undergo fractionations with no or negative effects on δ^13^C values^41,68–70^ in contrast to strongly positive fractionations usually associated with kinetic transformations. Newly produced microbial biomass will contain proteins derived from newly available carbon sources (e.g., sugars, short-chain acids, lipids) through cell hydrolysis or fermentation. Given that the relatively small negative fractionation observed for δ^13^C values in this study occurs equally across all tissue types and amino acid classes, independent of the wide range of lipid content between liver and collagen, the most likely driver is early colonization and proliferation of anaerobic microbes and the resulting microbial fermentation of sugars^69,71^ through the early stages of putrefaction.

### Decomposition Effects on Nitrogen

Examination of decomposition impact on bulk and amino acid stable isotope values solely indicates a marginal increase of 0.6‰ for glutamic acid δ^15^N values. This result contrasts with previous work that observed a wider range of small to large positive fractionations for δ^15^N values, commonly expected with the breakdown of amino acids, including decarboxylation and subsequent export of amines (e.g., putrescine, cadaverine). Clearly, increased values for bulk and amino acid nitrogen have been observed for burial and bacterial inoculation of mammal bones,^41,72^ likely reflecting the development of later successional stages of microbial consortia with well-adapted metabolism for using freely available substrate pools in anaerobic settings to support the breakdown and export of proteinaceous nitrogen. The lack of a uniform effect from decomposition observed in this study indicates that limited putrefaction has occurred in the early decomposition stages (e.g., 1 to 3) for harbor porpoise. Therefore, nitrogen stable isotope values predominantly and faithfully reflect the values that were incorporated prior to death through moderate decomposition, with the important exception of the marginal increase for glutamic acid. It is expected that decomposition will further change nitrogen values as skeletonization progresses, but bulk and amino acid δ^15^N values remained largely unaffected across the multiple tissue types examined here.

Terrestrial decomposition proceeds via a succession of microbial communities, with the quick development of consortia with unique metabolisms adept at breaking down amino acids and lipids.^73^ It should be expected that the stable isotope values of decomposing tissues will reflect the dominant microbial metabolic processes during microbial community succession, as well as the availability of substrates as putrefaction progresses. Early stages of decomposition in marine settings appear to predominantly reflect fermentative metabolisms, disproportionately impacting δ^13^C values, with later decomposition stages reflecting increased values for both carbon and nitrogen caused by kinetic fractionations associated with the mobilization of lipids, modification of proteins and their amino acids, and the export of nitrogenous compounds as putrefaction becomes thoroughly established.

### Tissue Type Differences

We found differences across tissue types and tissue-blood comparisons for bulk δ^13^C, δ^15^N, and δ^34^S values. Bone collagen has higher δ^13^C values and lower δ^34^S values across tissue types, reflecting differences likely arising from 1) a combination of biochemical pathways for the incorporation of dietary material into collagen, as well as 2) a slower turnover (1.5 to 2 years) versus other tissues compared against in this study (1 to 4 months). Mammal collagen has a limited composition of sulfur-containing compounds, as it does not contain cysteine,^74^ leaving methionine as the sole S source, with considerable differences in δ^34^S values previously observed between methionine and cysteine.^75^ Muscle tissue also has lower δ^15^N values, likely reflecting differing nitrogen requirements to support the turnover of skeletal musculature versus other tissues. Patterns of differences in both amino acid δ^13^C and δ^15^N values are different among amino acid classes, although differences are generally larger for nitrogen than for carbon values among tissue types. The multivariate analyses (ASCA) serve to further highlight differences observed among tissue types, with liver, liver fluid, and muscle grouping more closely together than collagen and blood for nitrogen and across the component 2 axis for carbon (Figure 6A). These differences arise as amino acids are incorporated into blood from digested dietary materials across the intestinal epithelial tissue and subsequently reworked and incorporated into liver, muscle, and bone collagen. Between-tissue differences in biochemical processing observed here are generally larger than those from the early stages of decomposition for both bulk and amino acid stable isotope values. Clear differences in essential, trophic, and source amino acid values (Figures 4A and 5A) further confirm that between-tissue differences in stable isotope values should be accounted for with tissue-specific trophic discrimination factors prior to applying multiple tissue types for the reconstruction of dietary time points across an organism’s lifetime.^21,76^ However, amino acid δ^13^C differences are smaller for essential than for nonessential or conditionally essential amino acid δ^13^C values, indicating that smaller adjustments are likely to be required to account for between-tissue differences for essential amino acids. Further work with controlled feeding studies to further deconvolute tissue discrimination factors from dietary changes across an animal’s lifetime is necessary to ensure accurate dietary reconstruction across a variety of tissue types.

### Liver Fluid as a Proxy

Liver fluid is potentially a proxy for blood in porpoises where decomposition has progressed beyond the point of being able to successfully retrieve a blood sample. Our analysis confirms that fluid sampled from the liver surface reflects a material that is more closely aligned with liver tissue than blood. The clear separation of confidence ellipsoids for both amino acid δ^13^C and δ^15^N values highlights the difference between the two materials’ compositions arising from differences across amino classifications (Figures 4A and 5A), but these relationships are less clear when examining differences in bulk stable isotope values. For bulk δ^15^N and δ^34^S values, liver fluid resembles blood, but for δ^13^C values, there is a clear shift toward liver values that would bias interpretations using liver fluid as a proxy. This points to a potentially useful proxy for some questions and applications, but one that requires consideration over whether the proxy is sufficiently separated from liver values to successfully inform outcomes, or whether the bias toward liver composition fundamentally skews outcomes enough to not be viable.

### Implications

Moderate decomposition produced small but consistent declines in bulk and amino acid δ^13^C values across tissues, likely reflecting microbial fermentation dominating early putrefaction, while δ^15^N values remained largely stable apart from glutamic acid. These effects were minor compared with between-tissue isotopic differences, emphasizing the need for tissue-specific discrimination factors when reconstructing dietary contributions across a consumer’s lifetime to reliably deconvolute dietary changes from metabolic differences among tissues. Liver fluid may provide a partial proxy for blood, though biases toward liver values warrant caution. Overall, early putrefaction introduces predictable shifts in δ^13^C values but preserves most nitrogen and sulfur isotopic values, allowing stable isotope data to retain ecological relevance across the initial decomposition stages.

## Supplementary Material



## Notes

Chromatogram examples for samples and standards, as well as details about the temperature ramps and settings for the gas chromatograph during analysis and statistical details supporting bulk and amino acid stable isotope results (PDF)

## #

Center for Applied Isotope Studies, University of Georgia, 120 Riverbend Road, Athens, GA, USA

## References

[1] Janaway, R. C. ; Percival, S. L. ; Wilson, A. S. Decomposition of Human Remains. In Microbiology and Aging: clinical Manifestations; Percival, S. L. ed., Humana Press: Totowa, NJ; 2009, pp. 313–334.

[2] Pringle, J. Further Experiments on Substances Resisting Putrefaction; With Experiments upon the Means of Hastening and Promoting It; By John Pringle M. D. F. R. S. The Royal Society 1749 491-496 1683–1775

[3] Harvey, G. The Vanities of Philosophy, and Physick; 2nd ed., Turner: London; 1700.

[4] Keenan S. W., DeBruyn J. M. (2019). Changes to vertebrate
tissue stable isotope (*δ*
^15^N) composition
during decomposition. Sci. Rep..

[5] Swann L. M., Forbes S. L., Lewis S. W. (2010). Analytical separations
of mammalian
decomposition products for forensic science: A review. Anal. Chim. Acta.

[6] Forbes, S. L. ; Perrault, K. A. ; Comstock, J. L. Microscopic post-mortem changes: the chemistry of decomposition Taphonomy Of Human Remains: forensic Analysis Of The Dead And The Depositional Environment 2017 26–38 10.1002/9781118953358.ch2

[7] Wheeler T. A., Kavanagh K. L., Daanen S. A. (2014). Terrestrial salmon carcass decomposition:
nutrient and isotopic dynamics in central Idaho. Northwest Sci..

[8] Troina G. C., Botta S., Dehairs F., Di Tullio J. C., Elskens M., Secchi E. R. (2020). Skin *δ*
^13^C and *δ*
^15^N reveal spatial
and temporal patterns of habitat and resource use by free-ranging
odontocetes from the southwestern Atlantic Ocean. Mar. Biol..

[9] Troina G. C., Riekenberg P., van der Meer M. T., Botta S., Dehairs F., Secchi E. R. (2021). Combining
isotopic analysis of bulk-skin and individual
amino acids to investigate the trophic position and foraging areas
of multiple cetacean species in the western South Atlantic. Environ. Res..

[10] Riekenberg P. M., Camalich J., Svensson E., IJsseldijk L. L., Brasseur S. M. J. M., Witbaard R., Leopold M. F., Rebolledo E. B., Middelburg J. J., van der Meer M. T. J. (2021). Reconstructing the diet,
trophic level and migration pattern of mysticete whales based on baleen
isotopic composition. R. Soc. Open Sci..

[11] Matsubayashi J., Osada Y., Tadokoro K., Abe Y., Yamaguchi A., Shirai K., Honda K., Yoshikawa C., Ogawa N. O., Ohkouchi N., Ishikawa N. F., Nagata T., Miyamoto H., Nishino S., Tayasu I. (2020). Tracking long-distance
migration of marine fishes using compound-specific stable isotope
analysis of amino acids. Ecol. Lett..

[12] Lamb A. L. (2016). Stable
isotope analysis of soft tissues from mummified human remains. Environ. Archaeol..

[13] Bartelink, E. J. ; Berg, G. E. ; Chesson, L. A. ; Tipple, B. J. ; Beasley, M. M. ; Prince-Buitenhuys, J. R. ; MacInnes, H. ; MacKinnon, A. T. ; Latham, K. E. Applications of stable isotope forensics for geolocating unidentified human remains from past conflict situations and large-scale humanitarian efforts. In New Perspectives in Forensic Human Skeletal Identification. Latham, K. E. ; Bartelink, E. J. ; Finnegan, M. pp. 175–184.Academic Press, 2018

[14] Lübcker N., Newsome S. D., Bester M. N., de Bruyn P. J. N. (2021). Validating the
use of bulk tissue stable isotope and amino acid *δ*
^15^N values measured in molted hair and epidermis of elephant
seals to assess temporal foraging niche specialization. Mar. Ecol.: Prog. Ser..

[15] Christianen M. J. A., Middelburg J. J., Holthuijsen S. J., Jouta J., Compton T. J., van der Heide T., Piersma T., Sinninghe Damsté J. S., van der
Veer H. W., Schouten S., Olff H. (2017). Benthic primary producers
are key to sustain the Wadden Sea food web: stable carbon isotope
analysis at landscape scale. Ecology.

[16] Larsen T., Taylor D. L., Leigh M. B., O’Brien D. M. (2009). Stable
isotope fingerprinting: a novel method for identifying plant, fungal,
or bacterial origins of amino acids. Ecology.

[17] Larsen T., Ventura M., Andersen N., O’Brien D. M., Piatkowski U., McCarthy M. D. (2013). Tracing carbon sources
through aquatic
and terrestrial food webs using amino acid stable isotope fingerprinting. PLoS One.

[18] Wada E., Mizutani H., Minagawa M. (1991). The use of stable isotopes
for food
web analysis. Crit. Rev. Food Sci. Nutr..

[19] Riekenberg P. M., van der Heide T., Holthuijsen S. J., van der Veer H. W., van der Meer M. T. J. (2022). Compound-specific stable isotope analysis of amino
acid nitrogen reveals detrital support of microphytobenthos in the
Dutch Wadden Sea benthic food web. Front. Ecol.
Evol..

[20] Minagawa M., Wada E. (1984). Stepwise enrichment of ^15^N along food chains: further
evidence and the relation between δ15N and animal age. Geochim. Cosmochim. Acta.

[21] Caut S., Angulo E., Courchamp F. (2009). Variation
in discrimination factors
(Δ15N and Δ13C): the effect of diet isotopic values and
applications for diet reconstruction. J. Appl.
Ecol..

[22] Koch, P. L. Isotopic study of the biology of modern and fossil vertebrates Stable Isotopes In Ecology And Environmental Science 2nd 2007 99–154 10.1002/9780470691854.ch5

[23] Szpak P. (2011). Fish bone
chemistry and ultrastructure: implications for taphonomy and stable
isotope analysis. J. Archaeol. Sci..

[24] Durante L. M., Sabadel A. J. M., Frew R. D., Ingram T., Wing S. R. (2020). Effects
of fixatives on stable isotopes of fish muscle tissue: implications
for trophic studies on preserved specimens. Ecol. Appl..

[25] Newsome S. D., Chivers S. J., Berman
Kowalewski M. (2018). The influence of lipid-extraction
and long-term DMSO preservation on carbon (*δ*
^13^C) and nitrogen (*δ*
^15^N) isotope values in cetacean skin. Mar. Mammal
Sci..

[26] Xu J., Yang Q., Zhang M., Zhang M., Xie P., Hansson L.-A. (2011). Preservation effects on stable isotope ratios and consequences
for the reconstruction of energetic pathways. Aquat. Ecol..

[27] Riekenberg P. M., Carney R. S., Fry B. (2018). Shell carbon isotope
indicators of
metabolic activity in the deep-sea mussel Bathymodiolus childressi. Deep Sea Res., Part I.

[28] Yurkowski D. J., Hussey A. J., Hussey N. E., Fisk A. T. (2017). Effects
of decomposition
on carbon and nitrogen stable isotope values of muscle tissue of varying
lipid content from three aquatic vertebrate species. Rapid Commun. Mass Spectrom.

[29] Miles, K. L. Forensic taphonomy and stable isotopes: an examination of δ^13^C and δ^15^N in decomposing skin, muscle, and grave soil as an indicator of post mortem interval; Dissertation, University of New Brunswick; 2024.

[30] Furuyama A., Yodo T., Funasaka N., Wakabayashi I., Oike T., Yoshioka M. (2020). Development of an analytical method
to exclude the effect of decomposition on carbon and nitrogen stable
isotope ratios using muscle samples collected from stranded narrow-ridged
finless porpoise (*Neophocaena asiaeorientalis*). Rapid Commun. Mass Spectrom..

[31] Frankel N. S., Vander Zanden H. B., Reich K. J., Williams K. L., Bjorndal K. A. (2012). Mother–offspring
stable isotope discrimination in loggerhead sea turtles *Caretta
caretta*. Endanger. Species Res..

[32] Ponsard S., Amlou M. (1999). Effects of several
preservation methods on the isotopic content of *Drosophila* samples. C R Acad. Sci.
III.

[33] Burrows D. G., Reichert W. L., Bradley
Hanson M. (2014). Effects of decomposition and storage
conditions on the *δ*
^13^C and *δ*
^15^N isotope values of killer whale (*Orcinus orca*) skin and blubber tissues. Mar. Mammal Sci..

[34] Horstmann-Dehn L., Follmann E. H., Rosa C., Zelensky G., George C. (2012). Stable carbon
and nitrogen isotope ratios in muscle and epidermis of arctic whales. Mar. Mammal Sci..

[35] Payo-Payo A., Ruiz B., Cardona L., Borrell A. (2013). Effect of tissue decomposition
on stable isotope signatures of striped dolphins *Stenella
coeruleoalba* and loggerhead sea turtles *Caretta caretta*. Aquat. Biol..

[36] Perkins M. J., Mak Y. K. Y., Tao L. S. R., Wong A. T. L., Yau J. K. C., Baker D. M., Leung K. M. Y. (2018). Short-term
tissue decomposition alters
stable isotope values and C: N ratio, but does not change relationships
between lipid content, C: N ratio, and *δ*
^13^C in marine animals. PLoS One.

[37] Cloyed C. S., Johnson C., DaCosta K. P., Clance L. R., Russell M. L., Díaz Clark C., Hieb E. E., Carmichael R. H. (2023). Effects
of tissue decomposition on stable isotope ratios and implications
for use of stranded animals in research. Ecosphere.

[38] Peiman K. S., Lin H.-Y., Power M., Hinch S. G., Patterson D. A., Cooke S. J. (2022). Effects of short-term
decomposition on isotope values
of fish tissues under natural conditions. Aquat.
Ecol..

[39] Goedkoop W., Åkerblom N., Demandt M. H. (2006). Trophic fractionation
of carbon and
nitrogen stable isotopes in *Chironomus riparius* reared
on food of aquatic and terrestrial origin. Freshwater
Biol..

[40] Turban-Just S., Schramm S. (1998). Stable carbon and nitrogen
isotope ratios of individual
amino acids give new insights into bone collagen degradation. Bull. Soc. Geol. Fr..

[41] Balzer A., Gleixner G., Grupe G., Schmidt H. L., Schramm S., Turban-Just S. (1997). In vitro decomposition of bone collagen by soil bacteria:
the implications for stable isotope analysis in archaeometry. Archaeometry.

[42] Hoke N., Rott A., Johler S., Reul A., Beck A., Günther A., Hochleitner R., Kaliwoda M., Harbeck M. (2019). How bone degradation,
age, and collagen extraction methods affect stable isotope analysis. Archaeol. Anthropol. Sci..

[43] Saul T. B., Chesson L. A., Steadman D. W., Gordon G. W. (2021). Considerations for
stable isotope analysis of human hair: the impact of postmortem environmental
exposure. Forensic Anthropol..

[44] Papastavrou V., Ryan C. (2023). Ethical standards for
research on marine mammals. Res. Ethics.

[45] Ryan C., Papastavrou V., Sand P. H. (2021). Ethical and legal considerations
for scientists collaborating with whalers: a case study of international
research using the outcome of contemporary whaling by Iceland. J. Int. Wildlife Law Policy.

[46] Pyenson N. D. (2010). Carcasses
on the coastline: measuring the ecological fidelity of the cetacean
stranding record in the eastern North Pacific Ocean. Paleobiology.

[47] Aarts G., Brasseur S., Poos J. J., Schop J., Kirkwood R., van Kooten T., Mul E., Reijnders P., Rijnsdorp A. D., Tulp I. (2019). Top-down pressure on a coastal ecosystem
by harbor seals. Ecosphere.

[48] Costa A. F., Botta S., Siciliano S., Giarrizzo T. (2020). Resource partitioning
among stranded aquatic mammals from Amazon and Northeastern coast
of Brazil revealed through carbon and nitrogen stable isotopes. Sci. Rep..

[49] Ijsseldijk, L. L. ; Brownlow, A. C. ; Mazzariol, S. European best practice on cetacean post-mortem investigation and tissue sampling; 2019. 10.31219/osf.io/zh4ra.

[50] Wilke J., Krause F., Niederer D., Engeroff T., Nürnberger F., Vogt L., Banzer W. (2015). Appraising the methodological quality
of cadaveric studies: validation of the QUACS scale. J. Anat..

[51] Collins M. J., Nielsen–Marsh C. M., Hiller J., Smith C. I., Roberts J. P., Prigodich R. V., Wess T. J., Csapò J., Millard A. R., Turner–Walker G. (2002). The survival of organic
matter in bone: a review. Archaeometry.

[52] Hedges R. E. M., Clement J. G., Thomas C. D. L., O’Connell T. C. (2007). Collagen
turnover in the adult femoral mid-shaft: Modeled from anthropogenic
radiocarbon tracer measurements. Am. J. Phys.
Anthropol..

[53] Matsubayashi J., Tayasu I. (2019). Collagen turnover and isotopic records in cortical
bone. J. Archaeol. Sci..

[54] Ijsseldijk L. L., ten Doeschate M. T. I., Brownlow A., Davison N. J., Deaville R., Galatius A., Gilles A., Haelters J., Jepson P. D., Keijl G. O. (2020). Spatiotemporal mortality and demographic trends
in a small cetacean: Strandings to inform conservation management. Biol. Conserv..

[55] Ijsseldijk L. L., Leopold M. F., Begeman L., Kik M. J. L., Wiersma L., Morell M., Rebolledo E. L. B., Jauniaux T., Heesterbeek H., Gröne A. (2022). Pathological
findings in stranded harbor porpoises
(*Phocoena phocoena*) with special focus on anthropogenic
causes. Front. Mar. Sci..

[56] Styring A. K., Kuhl A., Knowles T. D. J., Fraser R. A., Bogaard A., Evershed R. P. (2012). Practical considerations in the determination
of compound-specific
amino acid δ^15^N values in animal and plant tissues
by gas chromatography-combustion-isotope ratio mass spectrometry,
following derivatisation to their N-acetylisopropyl esters. Rapid Commun. Mass Spectrom..

[57] Chikaraishi Y., Kashiyama Y., Ogawa N. O., Kitazato H., Ohkouchi N. (2007). Metabolic
control of nitrogen isotope composition of amino acids in macroalgae
and gastropods: Implications for aquatic food web studies. Mar. Ecol.: Prog. Ser..

[58] Silverman S. N., Phillips A. A., Weiss G. M., Wilkes E. B., Eiler J. M., Sessions A. L. (2022). Practical considerations for amino acid isotope analysis. Org. Geochem..

[59] Riekenberg P. M., van der Meer M., Schouten S. (2020). Practical considerations for improved
reliability and precision during determination of δ15N values
in amino acids using a single combined oxidation–reduction
reactor. Rapid Commun. Mass Spectrom..

[60] Yarnes C. T., Herszage J. (2017). The relative influence of derivatization and normalization
procedures on the compound-specific stable isotope analysis of nitrogen
in amino acids. Rapid Commun. Mass Spectrom..

[61] Smilde A.
K., Jansen J. J., Hoefsloot H. C., Lamers R.-J. A., Van
Der Greef J., Timmerman M. E. (2005). ANOVA-simultaneous component analysis
(ASCA): a new tool for analyzing designed metabolomics data. Bioinformatics.

[62] Martin M., Govaerts B. (2020). LiMM-PCA: Combining
ASCA+ and linear mixed models to
analyse high-dimensional designed data. J. Chemom..

[63] Vane K., Cobain M. R. D., Larsen T. (2025). The power
and pitfalls of amino acid
carbon stable isotopes for tracing origin and use of basal resources
in food webs. Ecol. Monogr..

[64] Fry B., Carter J. F. (2019). Stable carbon isotope diagnostics of mammalian metabolism,
a high-resolution isotomics approach using amino acid carboxyl groups. PLoS One.

[65] DeNiro M. J., Epstein S. (1977). Mechanism of Carbon
Isotope Fractionation Associated
with Lipid Synthesis. Science.

[66] Javan G. T., Finley S. J., Smith T., Miller J., Wilkinson J. E. (2017). Cadaver
Thanatomicrobiome Signatures: The Ubiquitous Nature of Clostridium
Species in Human Decomposition. Front Microbiol..

[67] Can I., Javan G. T., Pozhitkov A. E., Noble P. A. (2014). Distinctive thanatomicrobiome
signatures found in the blood and internal organs of humans. J. Microbiol. Methods.

[200] Guo D., Lei J., He C., Peng Z., Liu R., Pan X., Meng J., Feng C., Xu L., Cheng Y., Chang M., Geng X. (2022). In vitro digestion
and fermentation
by human fecal microbiota of polysaccharides from Clitocybe squamulose. International journal of biological macromolecules.

[68] Zhang B.-L., Fallourd V., Role C., Martin G. J. (2003). Comparison of isotopic
fractionation in lactic acid and ethanol fermentations. Bioorg. Chem..

[69] Doering B. (2019). Effects of
fermentation on the carbon and nitrogen isotopes of Chinook salmon. J. Archaeol. Sci..

[70] Mueller E. P., Heuer V. B., Leadbetter J. R., Hinrichs K.-U., Sessions A. L. (2025). Metabolic
controls on the carbon isotope fractionations of bacterial fermentation. Proc. Natl. Acad. Sci. U. S. A..

[71] Penning H., Conrad R. (2006). Carbon isotope effects associated
with mixed-acid fermentation
of saccharides by *Clostridium papyrosolvens*. Geochim. Cosmochim. Acta.

[72] Christensen J. T., Richardson K. (2008). Stable isotope
evidence of long-term changes in the
North Sea food web structure. Mar. Ecol.: Prog.
Ser..

[73] Metcalf J.
L., Xu Z. Z., Weiss S., Lax S., Van Treuren W., Hyde E. R., Song S. J., Amir A., Larsen P., Sangwan N., Haarmann D., Humphrey G. C., Ackermann G., Thompson L. R., Lauber C., Bibat A., Nicholas C., Gebert M. J., Petrosino J. F., Reed S. C., Gilbert J. A., Lynne A. M., Bucheli S. R., Carter D. O., Knight R. (2016). Microbial
community assembly and metabolic function during mammalian corpse
decomposition. Science.

[74] Gauza-Włodarczyk M., Kubisz L., Włodarczyk D. (2017). Amino acid composition in determination
of collagen origin and assessment of physical factors effects. Int. J. Biol. Macromol..

[75] Phillips A. A., Wu F., Sessions A. L. (2021). Sulfur
isotope analysis of cysteine and methionine
via preparatory liquid chromatography and elemental analyzer isotope
ratio mass spectrometry. Rapid Commun. Mass
Spectrom..

[76] Stephens R. B., Ouimette A. P., Hobbie E. A., Rowe R. J. (2022). Reevaluating trophic
discrimination factors (*Δ*
^13^C and *Δ*
^15^N) for diet reconstruction. Ecol. Monogr..

