# Supplementary material for: Decomposition-Induced Change in Stranded Megafauna: Decoupling Postmortem Impacts from the Isotopic Niche
Source: Environ Sci Technol. 2026 Jul 10;60(29):20585–97. doi: 10.1021/acs.est.6c03636 (PMC13421979; doi:10.1021/acs.est.6c03636)
Supplement: Supplementary file 1 [file es6c03636_si_001.pdf]

Supplemental Material supporting:

Decomposition-induced change in stranded megafauna: Decoupling post-mortem impacts from the isotopic niche

Philip Riekenberg[1,†*], Lonneke L. IJsseldijk[2], Mardik Leopold[3], Andrea Gröne[2], Marcel T.J. van der Meer[1]

(1) Department of Marine Microbiology & Biogeochemistry, NIOZ Royal Netherlands Institute for Sea Research, PO Box 59, Den Hoorn, 1790AB, The Netherlands

(2) Division of Pathology, Department of Biomolecular Health Sciences, Faculty of Veterinary Medicine, Utrecht University, Yalelaan 1, 3854 CL, Utrecht, The Netherlands

(3) Wageningen Marine Research, Wageningen University and Research, Ankerpark 27, 1781 AG, Den Helder, The Netherlands

(†) Current address: Center for Applied Isotope Studies, University of Georgia, 120 Riverbend Road, Athens, GA, USA

*corresponding author: phrieken@gmail.com

Supplemental 1: GC-C-irMS ramp schedule and analysis details.

For C from amino acids, 1 µl of NAIP derivative diluted to target concentration in DCM based on GC-FID analysis is injected on column at 50°C and separated on a DB-5MS column (60m × 0.32mm × 0.5µm film thickness; Agilent) at a constant flow rate of 2.0 ml min^{-1} using the temperature program: 50°C for 1 min; ramp to 175°C at 50°C min^{-1} and hold for 3 min; ramp to 185°C at 3°C min^{-1} and hold for 2 min; then ramp to 310°C at 10°C min^{-1} and hold for 15 min. Each daily sequence begins with an oxidation of 30 min prior to analysis and is maintained with a 5 min seed oxidation run prior to each run. This analysis uses an ~1.5 m precolumn with daily maintenance of removing the portion of the precolumn seated in the injector to maximize peak intensity and to keep peaks from tailing. From this, we report $\delta^{13}C$ values for alanine, aspartic acid, glutamic acid, glycine, isoleucine, phenylalanine, proline, serine, threonine, tyrosine, and valine with precision of samples and standards of <±0.5‰. Lysine was not resolved sufficiently in standards or samples and methionine was sufficiently resolved in standards but did not consistently appear in samples at the target concentration used for this analysis. We used the same 15 amino acid 'offset' mix and 7 amino acid 'scaling' mix and the same daily sequence structure (10 offset and 2 scaling standards) that is presented in Riekenberg, et al. (2020).[67] with $\delta^{13}C$ values measured locally via elemental analyzer isotope ratio mass spectrometry and provided by Arndt Schimmelmann, Indiana University[77], respectively.

For $\delta^{15}N$ from amino acids, NPIP derivatives were resuspended in ethyl acetate to target concentration from GC-FID analysis and injected on column at 70°C and separated on a DB-5MS column (60m × 0.32mm × 0.5µm film thickness; Agilent) at a constant flow rate of 2.0 ml min^{-1} using the temperature program detailed in Riekenberg et al.[67]. Removing the portion of precolumn seated in the injector occurred every other day prior to the daily oxidation (2 h). Seed oxidation was 20 seconds after each run for standards and samples. From this analysis we report alanine, aspartic acid, glutamic acid, glycine, isoleucine, leucine, lysine, phenylalanine, serine, threonine, tyrosine, and valine with a precision of samples and standards of <0.5‰. Standards were sufficiently resolved for methionine but sample peaks did not consistently appear consistently in samples at the target concentration used for this analysis.

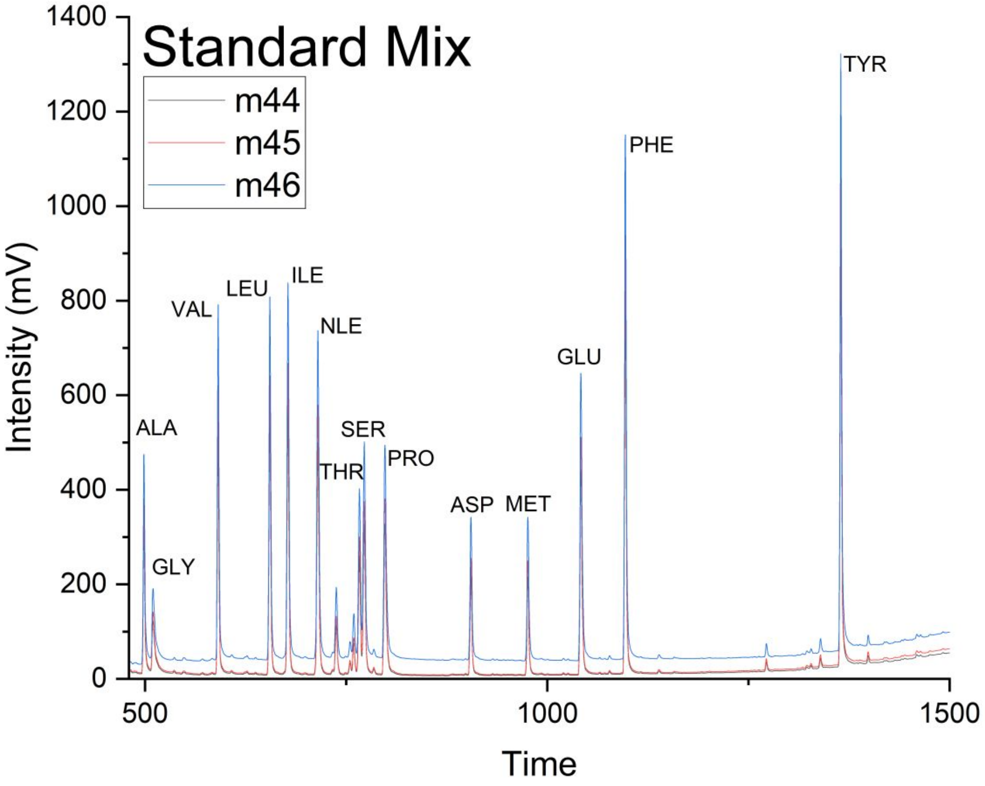

Supplemental Fig. 1: Chromatograph of the 15 amino acid standard mix used daily to standardize offsets for oxidation and the addition of 5 to 8 C from the NAIP derivatization.

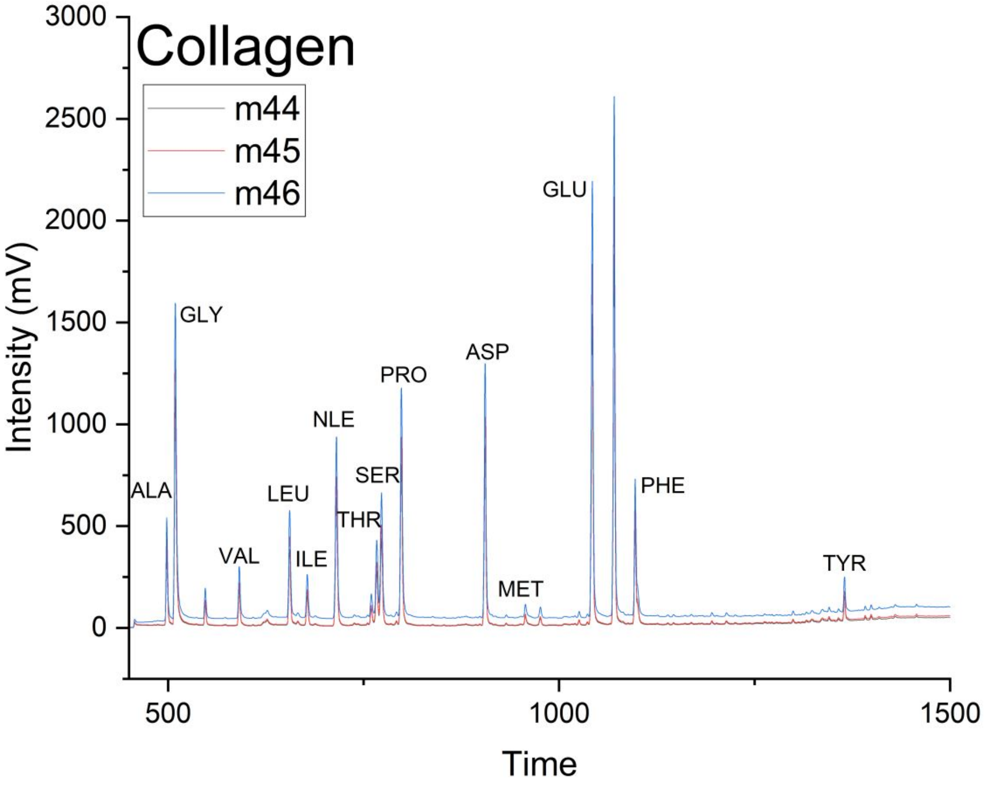

Supplemental Figure 2: Chromatograph of the 13 potential amino acids measured from collagen in this data set. Methionine is not reported due to being below the detection limit and lysine does not appear likely due to the relatively low polarity of the DB5MS column.

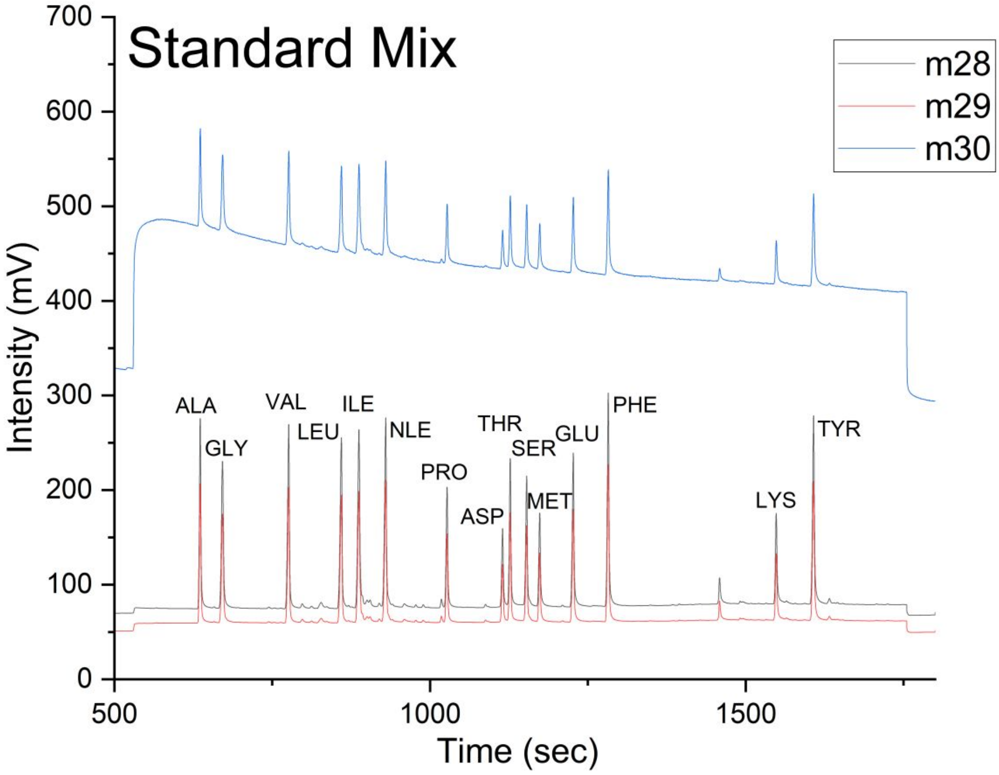

Supplemental Fig. 3: Chromatograph of the 15 amino acid standard mix used daily to standardize offsets for oxidation conditions from the reactor on each amino acid.

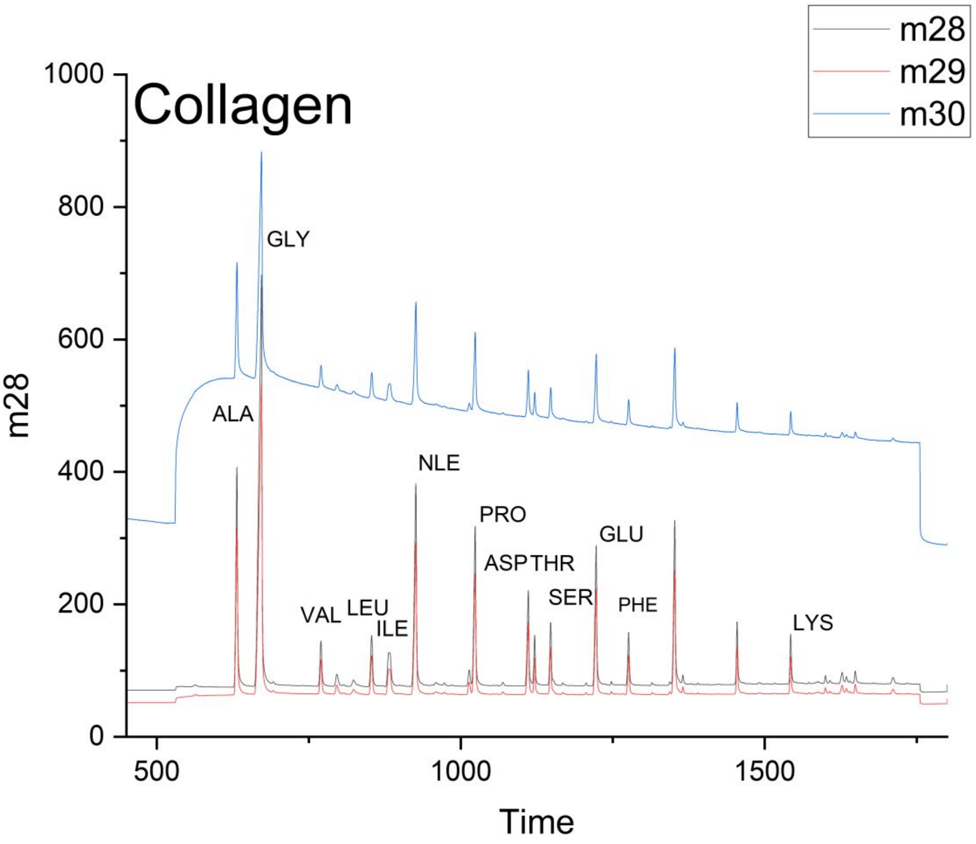

Supplemental Figure 4: Chromatograph of the 12 potential amino acids measured from collagen in this data set. Methionine and tyrosine are not reported due to being below the detection limit.

Supplemental 2: Results and statistical tables

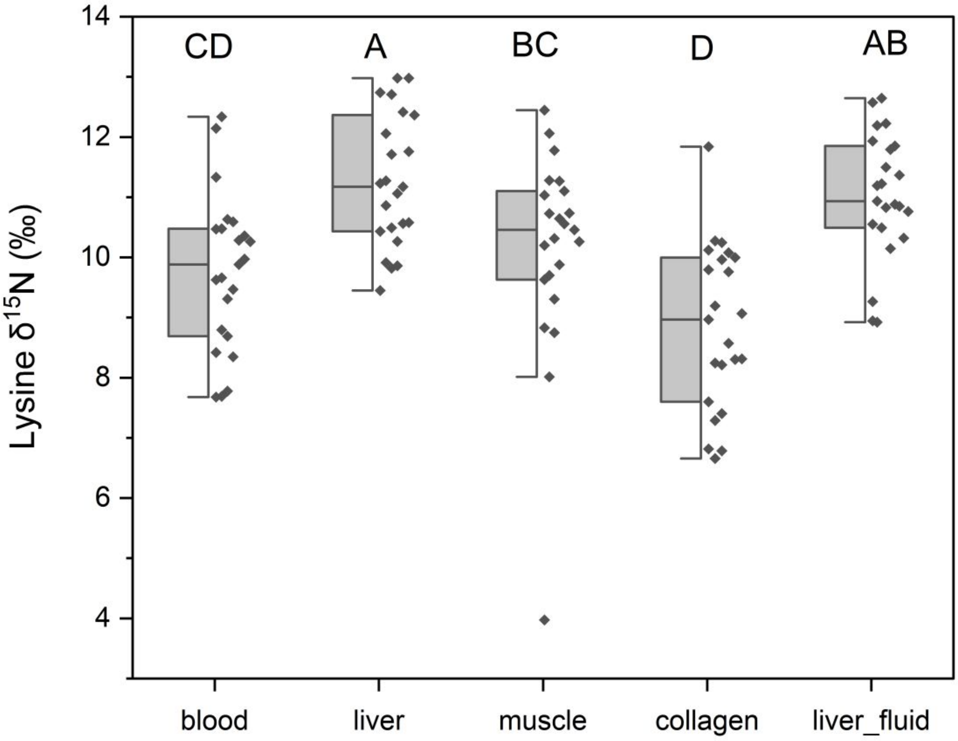

Supplemental Figure 5: Lysine $\delta^{15}N$ values across tissue types. Boxes represent the 25 to 75% quartiles, whiskers are the 1.5 interquartile range and the line indicates the median value for each grouping. Letters indicate likewise groupings from post hoc Tukey's honest significant difference at α=0.05 for comparison across and between tissue types for each normalized amino acid.

Supplemental Table 1: Bulk stable isotope statistical tests

Bulk stable isotope values

2-Way ANOVAs with Power Analysis

$\delta^{13}C$ (‰) values

	DF	Sum of Squares	Mean Square	F Value	P Value	
Tissue type	4	11.55536	2.88884	4.70348	0.00157	*
Decomposition Status	1	5.87158	5.87158	9.55983	0.00255	*
Interaction	4	0.36005	0.09001	0.14655	0.96416	
Model	9	20.13816	2.23757	3.64312	5.43E-04	*
Error	105	64.4902	0.61419			
Corrected Total	114	84.62836				

**Tissue Type**	Alpha	Sample Size	Power	
Actual Power	0.05	115	0.94288	*

**Decomposition Status**	Alpha	Sample Size	Power	
Actual Power	0.05	115	0.8651	*

δ^{15}N (‰) values

	DF	Sum of Squares	Mean Square	F Value	P Value	
Tissue type	4	32.58016	8.14504	8.23959	<0.0001	*
Decomposition Status	1	0.07226	0.07226	0.07309	0.78741	
Interaction	4	0.25472	0.06368	0.06442	0.99226	
Model	9	36.48907	4.05434	4.1014	1.54E-04	*
Error	105	103.79513	0.98853			
Corrected Total	114	140.2842				

**Tissue Type**	Alpha	Sample Size	Power	
Actual Power	0.05	115	0.99808	*

δ^{34}S (‰) values

	DF	Sum of Squares	Mean Square	F Value	P Value	
Tissue type	4	260.57333	65.14333	9.23578	<0.0001	*
decomp status	1	2.60118	2.60118	0.36879	0.54493	
Model	5	263.17451	52.6349	7.46238	<0.0001	*
Error	109	768.81692	7.05337			
Corrected Total	114	1031.99143				

**Tissue Type**	Alpha	Sample Size	Power	
Actual Power	0.05	115	0.99935	*

Stable istotope values for tissue type differences

2-Way ANOVAs with Power Analysis

Δ^{13}C (‰) values

	DF	Sum of Squares	Mean Square	F Value	P Value	
Tissue type	3	11.92958	3.97653	5.8189	0.00116	*
Decomposition Status	1	0.33487	0.33487	0.49002	0.48585	

Interaction	3	0.35848	0.11949	0.17486	0.91313	
Model	7	15.24442	2.17777	3.18676	0.00485	*
Error	84	57.40404	0.68338			
Corrected Total	91	72.64845				

**Tissue Type**	Alpha	Sample Size	Power	
Actual Power	0.05	92	0.94353	*

$\Delta^{15}N$ (‰) values

	DF	Sum of Squares	Mean Square	F Value	P Value	
Tissue type	3	32.30852	10.76951	9.85149	<0.0001	*
Decomposition Status	1	0.29219	0.29219	0.26728	0.60652	
Interaction	3	0.26182	0.08727	0.07983	0.97079	
Model	7	36.56927	5.22418	4.77886	1.47E-04	*
Error	84	91.82763	1.09319			
Corrected Total	91	128.3969				

**Tissue Type**	Alpha	Sample Size	Power	
Actual Power	0.05	92	0.99722	*

$\Delta^{34}S$ (‰) values

	DF	Sum of Squares	Mean Square	F Value	P Value	
Tissue type	3	211.8381	70.6127	6.70256	4.13E-04	*
Decomposition Status	1	1.03768	1.03768	0.0985	0.75442	
Interaction	3	12.28645	4.09548	0.38874	0.7614	
Model	7	231.6492	33.09274	3.14116	0.00537	*
Error	84	884.9557	10.53519			
Corrected Total	91	1116.605				

**Tissue Type**	Alpha	Sample Size	Power	
Actual Power	0.05	92	0.96944	*

Supplemental Table 2: Amino acid stable isotope statistical tests

Lysine amino acid N

2-Way ANOVAs
δ^{15}N AA (‰)
values

	DF	Sum of Squares	Mean Square	F Value	P Value	
tissue	4	86.84708	21.71177	12.28848	<0.0001	*
decomp	1	3.2704	3.2704	1.85099	0.17658	
Interaction	4	4.59348	1.14837	0.64996	0.62816	
Model	9	95.3388	10.5932	5.99557	<0.0001	*
Error	105	185.5181	1.76684			
Corrected Total	114	280.8569				

Lysine Normalized Amino Acid N

2-Way ANOVAs

δ^{15}N AA (‰) values

Glx-Lys

	DF	Sum of Squares	Mean Square	F Value	P Value	
tissue type	4	84.60308	21.15077	16.37663	<0.0001	*
decomp	1	9.96657	9.96657	7.71692	0.00649	*
Interaction	4	4.71776	1.17944	0.91322	0.45921	
Model	9	114.8231	12.75812	9.87836	<0.0001	
Error	104	134.3183	1.29152			
Corrected Total	113	249.1413				

Pro-Lys

	DF	Sum of Squares	Mean Square	F Value	P Value	
tissue type	4	343.3706	85.84265	10.09895	<0.0001	*
decomp	1	0.15697	0.15697	0.01847	0.89217	
Interaction	4	24.93384	6.23346	0.73333	0.57124	
Model	9	388.6166	43.17962	5.07986	<0.0001	*
Error	104	884.0158	8.50015			
Corrected Total	113	1272.632				

Phe-Lys

	DF	Sum of Squares	Mean Square	F Value	P Value	
tissue type	4	204.018	51.0045	27.73242	<0.0001	*
decomp	1	0.91244	0.91244	0.49612	0.48277	
Interaction	4	16.11141	4.02785	2.19004	0.0751	
Model	9	263.4292	29.26991	15.91478	<0.0001	*
Error	105	193.1124	1.83917			

Corrected Total	114	456.5415				

Gly-Lys

	DF	Sum of Squares	Mean Square	F Value	P Value	
tissue type	4	182.838	45.70951	14.7725	<0.0001	*
decomp	1	0.05723	0.05723	0.0185	0.89208	
Interaction	4	13.76568	3.44142	1.11221	0.35478	
Model	9	212.8342	23.64825	7.6427	<0.0001	*
Error	105	324.894	3.09423			
Corrected Total	114	537.7283				

Thr-Lys

	DF	Sum of Squares	Mean Square	F Value	P Value	
tissue type	4	181.735	45.43375	9.36044	<0.0001	*
decomp	1	1.65373	1.65373	0.34071	0.56067	
Interaction	4	7.54627	1.88657	0.38868	0.81633	
Model	9	195.7058	21.74508	4.48001	<0.0001	*
Error	105	509.6496	4.85381			
Corrected Total	114	705.3553				

73

AA N values for Lysine Normalized Tissues against Blood

2-Way ANOVAs

Δ^{15}N AA (‰) values

Glx-Lys

	DF	Sum of Squares	Mean Square	F Value	P Value	
tissue difference	3	83.70773	27.90258	14.0692	<0.0001	*
decomp state	1	0.28567	0.28567	0.14404	0.70525	
Interaction	3	10.96802	3.65601	1.84345	0.14553	
Model	7	116.2362	16.60517	8.37276	<0.0001	*
Error	84	166.592	1.98324			
Corrected Total	91	282.8281				

Pro-Lys

	DF	Sum of Squares	Mean Square	F Value	P Value	
tissue difference	3	521.0719	173.6906	10.33138	<0.0001	*
decomp state	1	0.01653	0.01653	9.84E-04	0.97505	
Model	4	521.0884	130.2721	7.74878	<0.0001	*
Error	87	1462.639	16.81195			

Corrected Total	91	1983.728				

Phe-Lys

	DF	Sum of Squares	Mean Square	F Value	P Value	
tissue difference	3	149.5592	49.85307	20.13568	<0.0001	*
decomp state	1	0.18309	0.18309	0.07395	0.78634	
Interaction	3	36.10699	12.03566	4.86121	0.00363	*
Model	7	249.4247	35.6321	14.39182	<0.0001	*
Error	84	207.972	2.47586			
Corrected Total	91	457.3967				

Gly-Lys

	DF	Sum of Squares	Mean Square	F Value	P Value	
tissue difference	3	97.79718	32.59906	5.37444	0.00196	*
decomp state	1	0.25425	0.25425	0.04192	0.83828	
Interaction	3	26.62382	8.87461	1.46311	0.23048	
Model	7	160.6252	22.94645	3.78306	0.0013	*
Error	84	509.5085	6.06558			
Corrected Total	91	670.1337				

Thr-Lys

	DF	Sum of Squares	Mean Square	F Value	P Value	
tissue difference	3	414.3789	138.1263	21.08503	<0.0001	*
decomp state	1	1.95459	1.95459	0.29837	0.58635	
Interaction	3	6.58575	2.19525	0.33511	0.79998	
Model	7	445.9158	63.70226	9.72417	<0.0001	*
Error	84	550.2771	6.55092			
Corrected Total	91	996.1929				

74

Mean centered amino acid C values

2-Way ANOVAs

$\delta^{13}C$ AA (‰) values

Leu_MC

	DF	Sum of Squares	Mean Square	F Value	P Value	
tissue type	4	16.6401	4.16003	2.44122	0.05131	**
decomp	1	8.86251	8.86251	5.20076	0.0246	*
Interaction	4	7.74463	1.93616	1.13619	0.34359	

	DF	Sum of Squares	Mean Square	F Value	P Value	
Model	9	32.20378	3.5782	2.09978	0.03583	*
Error	105	178.9283	1.70408			
Corrected Total	114	211.1321				

Phe_MC

	DF	Sum of Squares	Mean Square	F Value	P Value	
tissue type	4	29.87322	7.46831	4.01662	0.00452	*
decomp	1	14.06096	14.06096	7.5623	0.00702	*
Interaction	4	8.03356	2.00839	1.08016	0.37018	
Model	9	51.47154	5.71906	3.07584	0.00259	*
Error	105	195.2317	1.85935			
Corrected Total	114	246.7033				

Ala_MC

	DF	Sum of Squares	Mean Square	F Value	P Value	
tissue type	4	19.88188	4.97047	5.37811	5.59E-04	*
decomp	1	7.00864	7.00864	7.58343	0.00694	*
Interaction	4	3.26106	0.81526	0.88213	0.47737	
Model	9	27.57499	3.06389	3.31517	0.00134	*
Error	105	97.04137	0.9242			
Corrected Total	114	124.6164				

Asp_MC

	DF	Sum of Squares	Mean Square	F Value	P Value	
tissue type	4	28.24438	7.06109	5.05056	9.21E-04	*
decomp	1	15.80461	15.80461	11.3045	0.00108	*
Interaction	4	5.76755	1.44189	1.03133	0.39468	
Model	9	46.66751	5.18528	3.70885	4.53E-04	*
Error	105	146.7985	1.39808			
Corrected Total	114	193.4661				

Glu_MC

	DF	Sum of Squares	Mean Square	F Value	P Value	
tissue type	4	23.92632	5.98158	2.99798	0.02184	*
decomp	1	14.51881	14.51881	7.27687	0.00814	*
Interaction	4	7.53494	1.88373	0.94413	0.44158	
Model	9	45.74247	5.0825	2.54736	0.01095	*
Error	105	209.4961	1.9952			
Corrected Total	114	255.2386				

Pro_MC

	DF	Sum of Squares	Mean Square	F Value	P Value	
tissue type	4	35.50602	8.8765	5.46598	4.89E-04	*
decomp	1	14.65138	14.65138	9.02204	0.00334	*
Interaction	4	5.11391	1.27848	0.78726	0.53597	
Model	9	60.59811	6.73312	4.14613	1.36E-04	*
Error	105	170.5152	1.62395			
Corrected Total	114	231.1133				

AA C values for mean Normalized Tissues against Blood

2-Way ANOVAs
$\Delta^{13}C$ AA (‰)
values

Leu_MC

	DF	Sum of Squares	Mean Square	F Value	P Value	
Tissue Type	3	37.23746	12.41249	7.57067	1.53E-04	*
Decomp	1	0.18465	0.18465	0.11262	0.73802	
Interaction	3	5.71844	1.90615	1.1626	0.32896	
Model	7	38.86946	5.55278	3.38677	0.00311	*
Error	84	137.7221	1.63955			
Corrected Total	91	176.5916				

Phe_MC

	DF	Sum of Squares	Mean Square	F Value	P Value	
Tissue Type	3	49.06843	16.35614	5.21272	0.00238	*
Decomp	1	2.09635	2.09635	0.66811	0.41602	
Interaction	3	14.90656	4.96885	1.58358	0.19942	
Model	7	67.34839	9.6212	3.06628	0.00633	*
Error	84	263.5701	3.13774			
Corrected Total	91	330.9185				

Ala_MC

	DF	Sum of Squares	Mean Square	F Value	P Value	
Tissue Type	3	37.23746	12.41249	7.57067	1.53E-04	*
Decomp	1	0.18465	0.18465	0.11262	0.73802	
Interaction	3	5.71844	1.90615	1.1626	0.32896	

Model	7	38.86946	5.55278	3.38677	0.00311	*
Error	84	137.7221	1.63955			
Corrected Total	91	176.5916				

Asp_MC

	DF	Sum of Squares	Mean Square	F Value	P Value	
Tissue Type	3	66.82361	22.27454	10.35143	<0.0001	*
Decomp	1	0.73964	0.73964	0.34372	0.55926	
Interaction	3	16.70822	5.56941	2.58822	0.05833	
Model	7	74.37891	10.62556	4.93791	1.04E-04	*
Error	84	180.7539	2.15183			
Corrected Total	91	255.1328				

Glu_MC

	DF	Sum of Squares	Mean Square	F Value	P Value	
Tissue Type	3	49.86533	16.62178	4.59504	0.005	*
Decomp	1	0.6362	0.6362	0.17587	0.67601	
Interaction	3	22.37837	7.45946	2.06214	0.1114	
Model	7	68.22788	9.74684	2.69448	0.01439	*
Error	84	303.856	3.61733			
Corrected Total	91	372.0839				

Pro_MC

	DF	Sum of Squares	Mean Square	F Value	P Value	
Tissue Type	3	68.00218	22.66739	8.34966	<0.0001	*
Decomp	1	1.95553	1.95553	0.72033	0.39845	
Interaction	3	7.6575	2.5525	0.94023	0.42503	
Model	7	91.23939	13.0342	4.80122	1.40E-04	*
Error	84	228.0406	2.71477			
Corrected Total	91	319.2799				

ANOVA Simultaneous Component Analysis

AA δ^{15}N- lysine normalized

AA δ^{15}N- Lysine normalized	Sum Squared	Variance explained	P value	
Tissue Type	1093.77	33.96	0	*
Decomposition status	12.92	0.4	0.49	
Tiss.Type:Decomp	64.77	2.01	0.49	
Residuals	2049.59	63.63	NA	

	Comp 1	Comp 2
Phe	-0.6	-0.18
Gly	-0.58	0.08
Pro	-0.35	0.78
Glu	-0.34	-0.23
Thr	-0.24	-0.55

AA δ^{13}C- mean normalized

	Sum Squared	Variance explained	P value	
Tissue Type	151.9	12.03	0	*
Decomposition status	74.91	5.93	0	*
Tiss.Type:Decomp	37.46	2.97	0.32	
Residuals	998.01	79.06	NA	

	Comp 1	Comp 2
Pro	0.53	-0.32
Phe	0.5	-0.28
Ala	0.43	-0.05
Asp	0.37	0.55
Leu	0.36	0.06
Glu	0.15	0.72
